# Large-scale mRNA transfer between *Haloxylon ammodendron* (Chenopodiaceae) and herbaceous root holoparasite *Cistanche deserticola* (Orobanchaceae)

**DOI:** 10.1016/j.isci.2022.105880

**Published:** 2022-12-27

**Authors:** Yanyan Fan, Qiqi Zhao, Huimin Duan, Shuxin Bi, Xiaomin Hao, Rui Xu, Runyao Bai, Ruonan Yu, Wenting Lu, Tiejun Bao, Hada Wuriyanghan

**Affiliations:** 1 Key Laboratory of Forage and Endemic Crop Biology, Ministry of Education, School of Life Sciences, Inner Mongolia University, Hohhot 010070, China; 2 Key Laboratory of Ecology and Resource Use of the Mongolian Plateau, Ministry of Education, School of Ecology and Environment, Inner Mongolia University, Hohhot 010021, China

**Keywords:** Plant biology, Interaction of plants with organisms, Omics, Transcriptomics

## Abstract

Exchanges of mRNA were shown between host and stem parasites but not root parasites. *Cistanche deserticola* (Orobanchaceae) is a holoparasitic herb which parasitizes on the roots of woody plant *Haloxylon ammodendron* (Chenopodiaceae). We used transcriptome sequencing and bioinformatic analyses to identify nearly ten thousand mobile mRNAs. Transcript abundance appears to be a driving force for transfer event and mRNA exchanges occur through haustorial junction. Mobility of selected mRNAs was confirmed *in situ* and in sunflower-*Orobanche cumana* heterologous parasitic system. Four *C*. *deserticola* →*H*. *ammodendron* mobile mRNAs appear to facilitate haustorium development. Of interest, two mobile mRNAs of putative resistance genes *CdNLR1* and *CdNLR2* cause root-specific hypersensitive response and retard parasite development, which might contribute to parasitic equilibrium. The present study provides evidence for the large-scale mRNA transfer event between a woody host and a root parasite, and demonstrates the functional relevance of six *C*. *deserticola* genes in host-parasite interactions.

## Highlights

• Ten thousand mRNAs transfer between *Haloxylon ammodendron* and root parasitic *Cistanche deserticola*
• RNA mobility and functional analysis were performed in sunflower-*Orobanche cumana* system
• *CdNLR1* and *CdNLR2* would cause root-specific HR and affect parasitic equilibrium

## Graphical abstract

**Figure:**
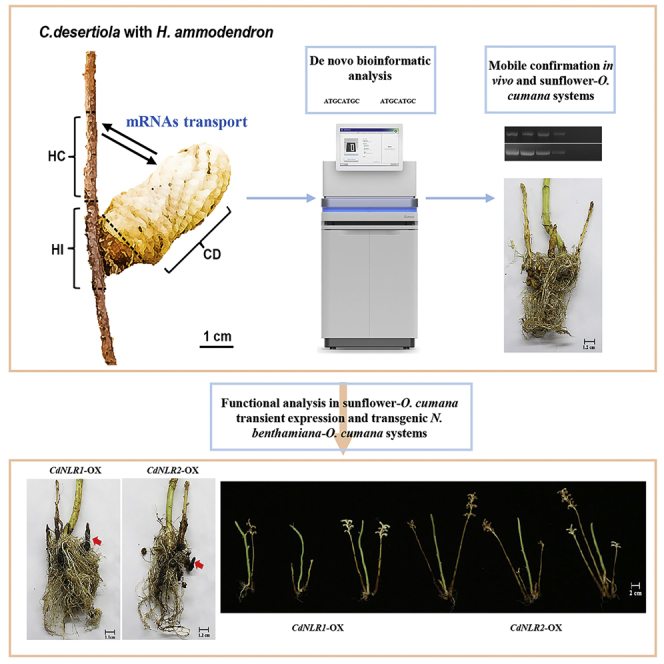

## Introduction

Increasing evidence suggests the importance of macromolecules such as proteins and mRNAs in both short- and long-distance communication in plants.^1^ Plasmodesmata are considered as short-distance transport channels, whereas vascular system carries out long-distance molecular transport between distal tissues.^2^^,^^3^ mRNA was reported to transfer in phloem.^4^ Recent studies have shown that the stem-loop structure of tRNA origin endowed mRNA with long-distance transport capacity.^5^ AtRRP44A, a subunit of the RNA exosome, was reported to interact with plasmodesmata and to mediate the cell-to-cell trafficking of KNOTTED1 (KN1) mRNA.^6^

Parasitic plants account for about 1% of flowering plants. The most important feature of parasitic plants is a specialized structure called haustorium, which establishes physical and physiological connection channels between the host and parasitic plants and so dominates most of their interactions.^7^ Parasitic plants rely on the host to sustain their growth, absorbing water and the nutrients such as photosynthates, amino acids and other intermediate metabolites from the host through the haustorium.^8^ Haustoria also function as the connection bridge between the hosts to transmit signaling molecule such as herbivore signal.^9^ It was also shown that the biomolecules including proteins and RNAs exchange bidirectionally through host-parasite connections. The earliest example of RNA transfer between the host and parasitic plants is the transmission of RNA virus from the infected host to the uninfected plant through dodder.^10^ Small RNAs (sRNAs) such as small interference RNA (siRNA) and microRNA (miRNA) were shown to move between host-parasite to regulate the target gene expression in recipient organisms.^11^^,^^12^^,^^13^ As for mRNA transfer, mobile mRNAs from the hosts tomato, pumpkin and alfalfa to the parasite *Cuscuta chinensis* have been demonstrated for several genes in the initial test.^14^ Through microarray analysis, Roney et al. found that 474 mRNAs were transferred from tomato to dodder.^14^ However, large-scale identification of transfer mRNAs was achieved only on the uses of next-generation sequencing technology. Kim et al. used transcriptome sequencing technology to identify large-scale and bidirectional mRNA transfer between the parasite *Cuscuta pentagona* and the hosts such as *Arabidopsis thaliana* and tomato.^15^ But the functional relevance of transfer mRNAs in host-parasite interactions was not clear.

Orobanchaceae is the largest parasitic angiosperm family in which many are facultative or obligate root parasites.^16^ *Cistanche* is a worldwide genus of holoparasitic desert plants in Orobanchaceae. *C*. *deserticola* is a holoparasite which parasitizes on the roots of a woody psammophyte, *Haloxylon ammodendron* (Chenopodiaceae).^17^ It was shown that chloroplast *rpoC2* gene was transferred from *H*. *ammodendron* to *C*. *deserticola* by horizontal gene transfer.^18^ Here, we identified nearly ten thousand mobile mRNAs between *C*. *deserticola* and *H*. *ammodendron* through next-generation transcriptome sequencing and bioinformatic analysis. The mobile mRNAs were ascertained by multiple sequence alignment, PCR validation, and especially utilization of a sunflower-*Orobanche cumana* parasitic system. The function of mobile mRNAs was also demonstrated for several genes by this heterologous parasitic system. To date, the reports on functional mRNAs exchange between host and parasite were very limited. Therefore, our study provides new insights into the parasitic mechanism from the view point of mobile mRNAs.

## Results

### Identification of mobile mRNAs between *C*. *deserticola* and *H*. *ammodendron*

**Figure 1:**
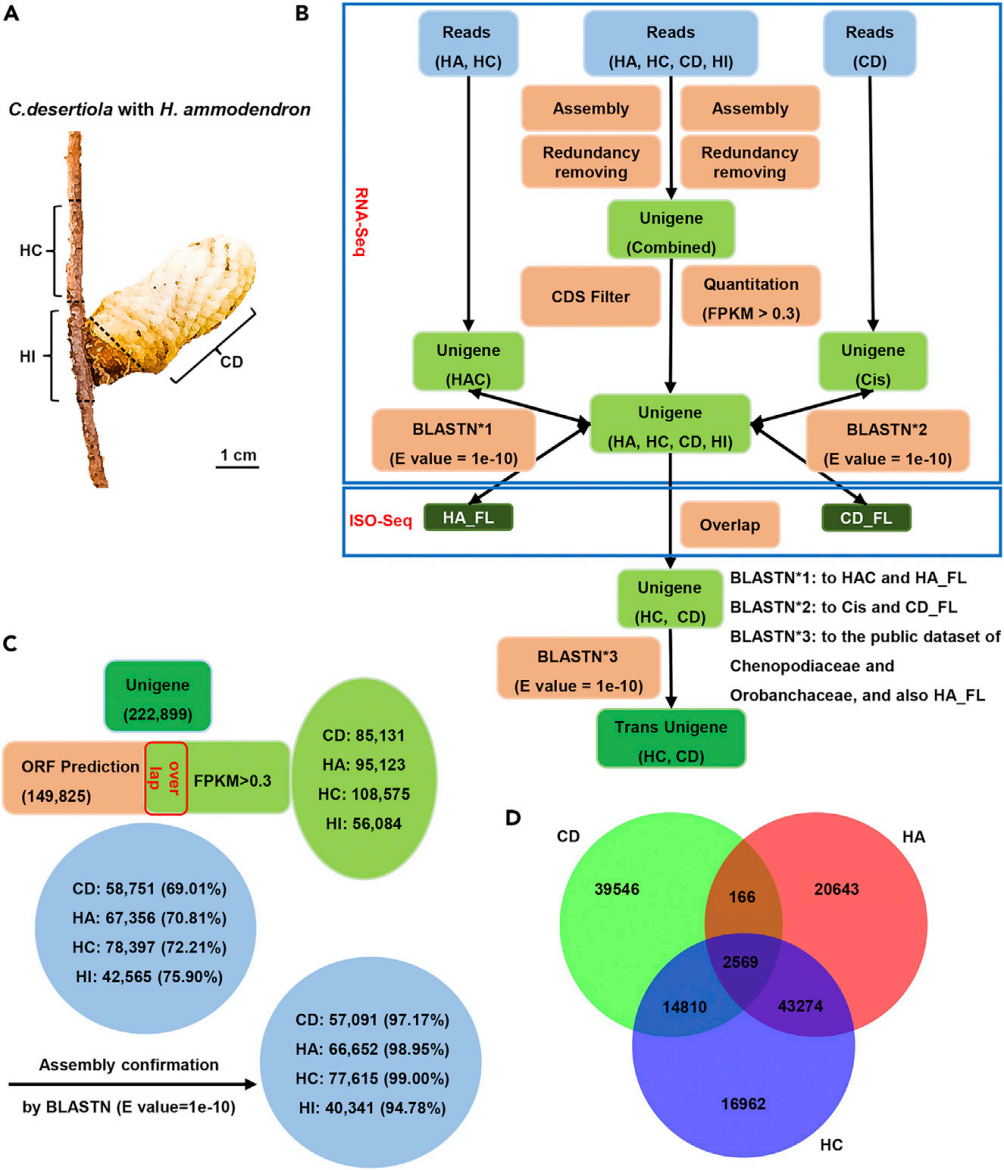
RNA-seq analysis and confirmation of mobile RNAs (A) Illustration of select tissues for RNA-seq analysis. CD, fresh succulent stems of *C*. *deserticola* near haustorial junction; HC, the roots of CD-infested *H*. *ammodendron*; HI, haustorial interface. Scale bars are indicated. (B) Outline of the strategies for sequence assembly and confirmation of mobile RNAs. RNA-seq, next generation RNA sequencing. ISO-Seq, full-length transcriptome sequencing. HA, the roots of non-parasitized *H*. *ammodendron*. HA.FL_NR, full-length transcriptome sequence of *H*. *ammodendron*. CD.FL_NR, full-length transcriptome sequence of *C*. *deserticola*. (C) ORF prediction and further confirmation of assembly result. ORF finder (https://www.ncbi.nlm.nih.gov/orffinder/) was applied for ORF prediction, and above assembled unigenes with the expression threshold value of (FPKM≥0.3) were cross-checked. The overlapped unigenes were further filtered with dual BLASTN (E value = 1e^−10^) against Cis and HAC unigenes. (D) Venn diagrams showing common and differed sets of transcripts between CD, HA and HC samples.

Tight physical connection of host *H*. *ammodendron* root and parasite *C*. *deserticola* makes it impossible to separate them at the haustorium. Therefore, we combined high-throughput RNA-sequencing (RNA-seq) and stepwise bioinformatic classification to identify the mobile transcripts between host and parasite. Four different set of samples were collected for RNA-seq analyses on Illumina HiSeq2500 platform. These include the root tissue of parasite-free host *H*. *ammodendron* (HA), succulent stem of *C*. *deserticola* (CD), the root tissue of host *H*. *ammodendron* parasitized with *C*. *deserticola* (HC), and the haustorial interface (HI). For HC and CD samples, the respective host root and parasite stem were collected 1 cm away from the parasitic junction (Figure 1A).

Reliable identification of source tissue for a given transcript from interacting organisms is a challenging issue. Therefore Ikeue et al. have developed a useful bioinformatic method to distinguish host and parasite transcripts.^19^ Here we used a modified bioinformatic approach which is based on their method to identify the mobile mRNAs between *C*. *deserticola* and *H*. *ammodendron* (Figure 1B). A grand total of 723,382,940 clean reads were generated from above libraries (Table S2), and these reads were assembled via different strategies (Figure 1B). As a result, all ten samples were hybrid assembled into 222,899 unigenes (termed “Combined” hereafter, Table S3). *C*. *deserticola* and *H*. *ammodendron* samples were also assembled into 107,752 and 194,720 unigenes respectively (termed “Cis” and “HAC” hereafter, Table S3). To visualize the variation as well as the similarity for all samples, we performed a principal component analysis (PCA) on the normalized FPKM values of all the detected genes in “Combined unigenes”. The PCA plot showed that the data for three biological replicates were clustered closely and were separated in different samples, especially between *H*. *ammodendron* and *C*. *deserticola* (Figure S1). Meanwhile, ORF prediction showed that 149,825 (67.23%) of the “Combined” unigenes were assumed to encode putative proteins (Figure 1C). The individually assembled unigenes with the expression threshold value of FPKM≥0.3 from four different samples were cross-checked with the ORF prediction results (Tables S4–S6). This analysis identified that 69.01–75.90% of the unigenes from the four samples CD, HC, HA and HI had putative ORF (Figure 1C). Full-length transcriptome sequencing was carried out and the data, CD_FL for *C*. *deserticola* and HA_FL for *H*. *ammodendron*, were used for assembly error correction for both host *H*. *ammodendron* and parasite *C*. *deserticola* as both species lack genomic data (Tables S8–S11). When the assigned unigenes from above four samples were filtered with dual BLASTN (E value = 1e^−10^) against Cis, HAC, CD_FL and HA_FL, 94.78–99.00% of them had reliable consensus sequences (Figure 1C and Table S7). Based on the above analysis, 17,379 (HA-exclusive 14,810 and HA-containing 2,569) common unigenes were finally retrieved from HC and CD on Venn diagram analysis of the unigenes from CD, HC and HA (Figure 1D). Among them, the former 14,810 unigenes are most probably of parasite CD origin because of their absence in HA, but a proportion of them might also represent HC→CD mobile mRNAs which are present only in HC but not in HA owing to their up-regulation on parasite attachment. The latter 2,569 unigenes most probably represent unidirectional HC→CD transfer mRNAs as they are also present in HA. The other unigenes were excluded from candidate mobile mRNAs as they were not shared between HC and CD. One should also note that 14,810 common unigenes in [HC, CD] outnumbered 166 common unigenes in [HA, CD] by 89 times, highly suggesting the reliability of our analyses as the latter was logically impossible and was caused by the inevitable errors from unigene assembly or bioinformatic analyses (Figure 1D).

**Figure 2:**
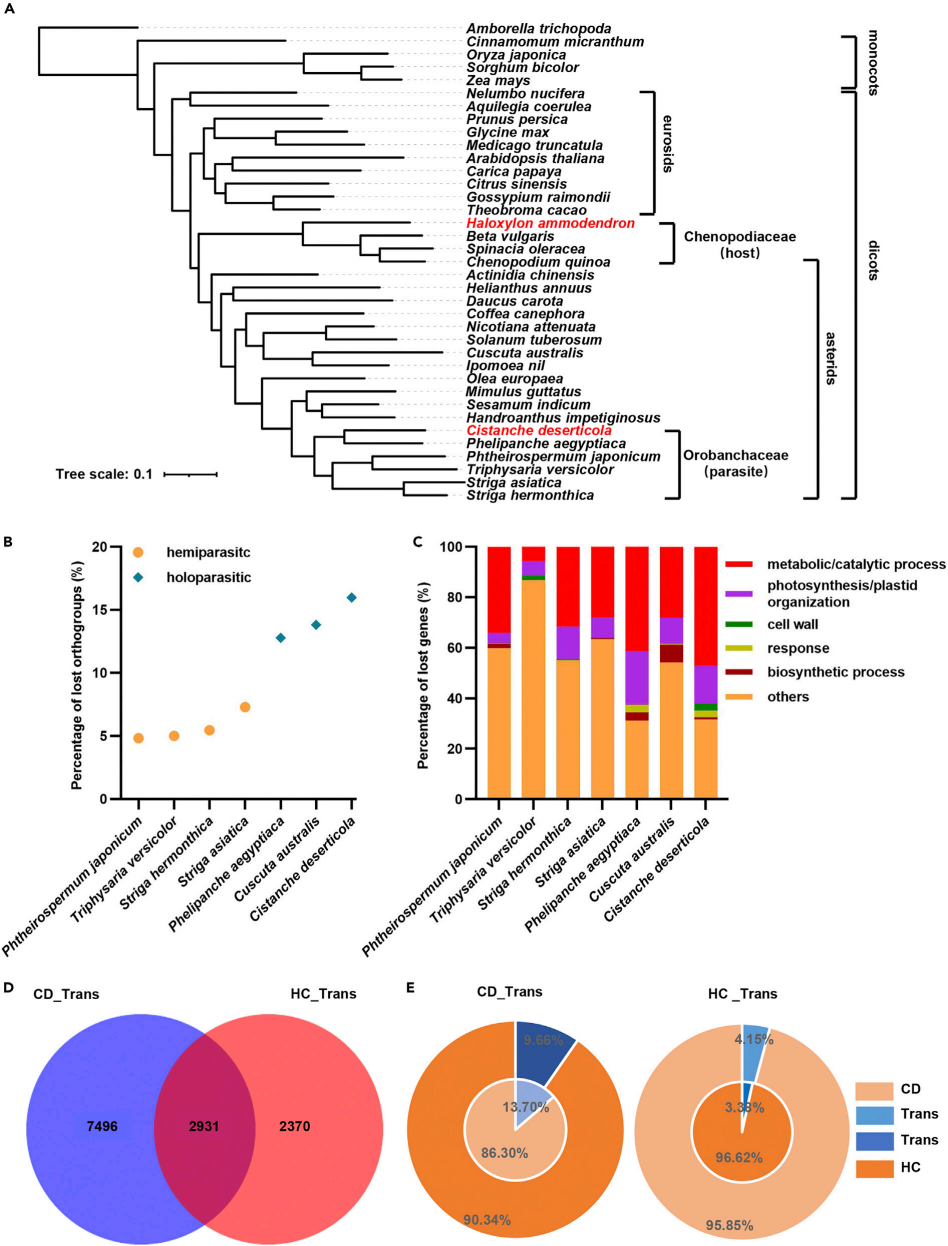
Gene loss analysis and further confirmation of mobile RNAs via multiple alignment (A) Phylogeny of flowering plant genomes. OrthoFinder was used to analyze the evolution of all protein sequences of 37 species, and to find the orthogonal group including direct homologous genes and collateral homologous genes among species. (B) Percentage of lost orthogroups. The number of orthogroup deletions in semi parasitic plants and total parasitic plants. (C) Percentage of lost unigenes. The proportion of genes related to photosynthesis and metabolism was shown. (D) Venn diagrams showing common and differed sets of transcripts between CD and HC samples on multiple alignment. CD_*trans*, CD→HC mobile RNAs. HC_*trans*, HC→CD mobile RNAs. (E) Pie charts show the proportions of mobile reads mapped to CD (outer circle) and HC (inner circle) transcriptomes.

The origin of putative mobile mRNAs was further confirmed via dual BLAST analyses to assign their sequence origin (Figure 1B and Table S12). Because of the lack of genomic data for both host and parasite, we utilized available sequence data for Chenopodiaceae and Orobanchaceae family organisms to which *H*. *ammodendron* and *C*. *deserticola* belong respectively. Our hypothesis is that mobile mRNAs of *H*. *ammodendron* origin most probably have higher homologies with their orthologs from other Chenopodiaceae species than those from Orobanchaceae species, while it is vice versa for mobile mRNA of *C*. *deserticola* origin, and the conserved orthologs probably have high similarity with both Orobanchaceae and Chenopodiaceae. This analysis could help us to ascertain the origins of the mobile mRNAs as they could be assigned to family level via homology searching. To prove our hypothesis, phylogenetic tree of thirty-seven species, including six Orobanchaceae and four Chenopodiaceae species, was constructed using OrthoFinder. The far evolutionary distance between Orobanchaceae and Chenopodiaceae species ascertained the reliability of our hypothesis (Figure 2A and Table S13). Gene loss analysis showed that the transcripts for many orthogroups and photosynthesis related genes were absent in *C*. *deserticola* (Figures 2B and 2C, Table S14, Data S2 and S3). Moreover, the Venn diagram analysis between *C*. *deserticola* and three other sequenced parasitic plants also showed that 53.84% (926/1,720) of the missing orthogroups in *C*. *deserticola* were also absent in other three parasitic plants, namely 50.81% (755/1486), 48.09% (377/784) and 50.00% (260/520) in *C*. *australis*, *Striga asiatica* and *Phtheirospermum japonicum* respectively (Figure S2). These results not only indicated the parasitic property of this species but also the reliability of unigene assembly from our analyses, and also supported our hypothesis to use relative plant species for confirmation of sequence origin. Therefore, the candidate transfer unigenes from above analyses were separated with dual BLASTN (E value = 1e^−10^) against a collection of public available sequence datasets from Orobanchaceae (transcriptome of *Triphysaria versicolor*, *Striga hermonthica and Phelipanche aegyptiaca*; EST and mRNA sequences from NCBI) and Chenopodiaceae (next-generation and full-length transcriptome of *H*. *ammodendron*; EST and mRNA sequences from NCBI) (Figure 1B and Table S12). As a result, 7,496 unigenes were confirmed to originate from the parasite *C*. *deserticola*, accounting for 9.66% (7,496/77,615) and 13.70% (7,496/54,721) of the unigenes in destination HC samples and source CD, respectively; and 2,370 unigenes were assigned to host *H*. *ammodendron*, accounting for 3.38% (2,370/70,119) and 4.15% (2,370/57,091) of the unigenes in source HC and destination CD samples, respectively (Figures 2D and 2E, Tables S7, S12, and S15). The common 2,931 unigenes were too homologous to be assigned to the exact source organism. These results indicated that nearly ten thousand (7,496 + 2,370 = 9,866) unigenes could transfer between root parasitic plant *C*. *deserticola* and woody plant host *H*. *ammodendron*. Much more (7,496/2,370 = 3.16-folds) mobile RNAs were of parasite *C*. *deserticola* origin than of host *H*. *ammodendron* origin, showing the mRNA transfer bias in a parasite→host direction.

### General properties of mobile mRNAs

**Figure 3:**
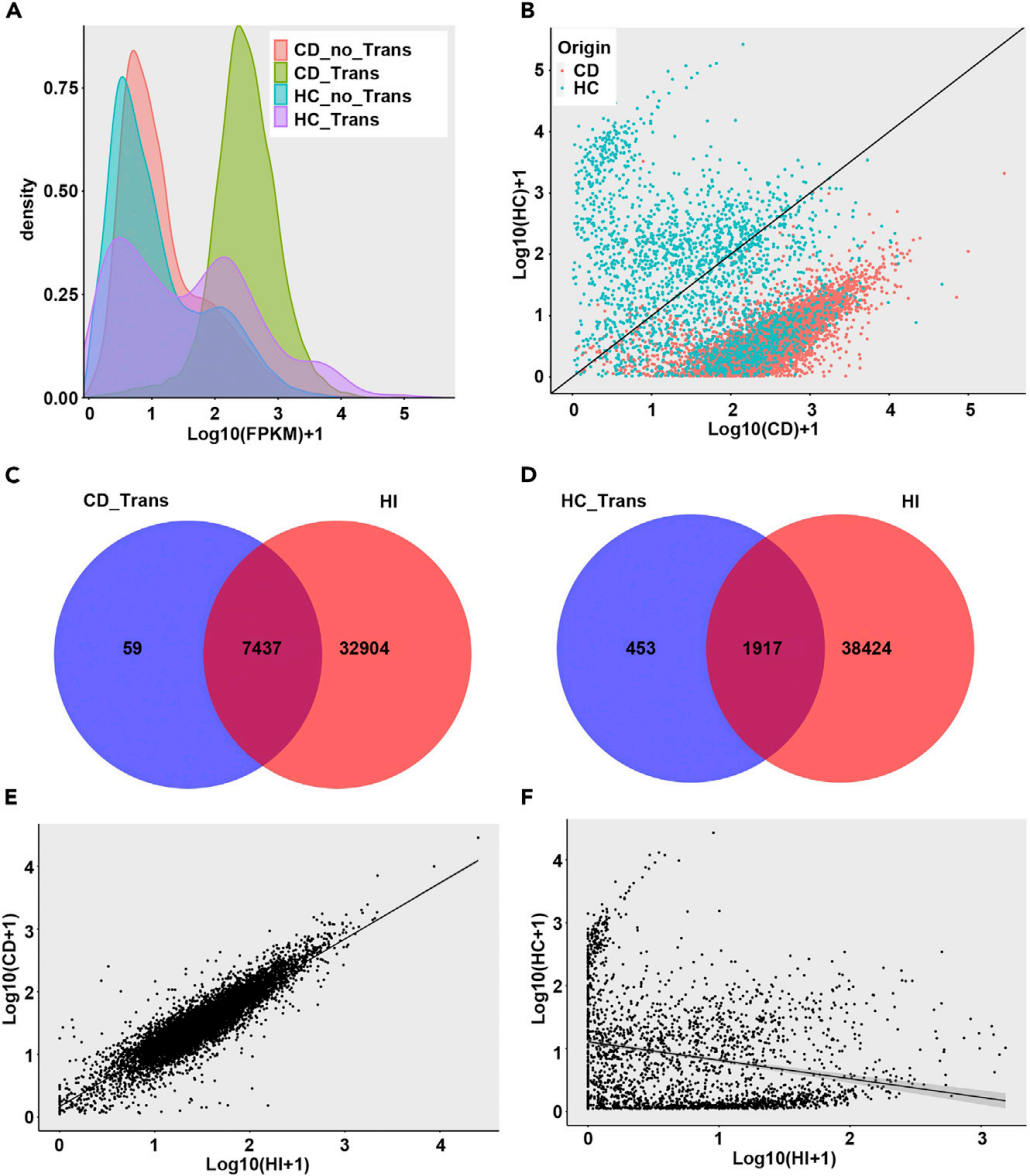
General properties of transfer and non-mobile RNAs (A) Distribution of unigene expression levels in source tissues. CD_no_*trans*, non-mobile RNAs from CD. CD_*trans*, mobile RNAs from CD. HC_no_*trans*, non-mobile RNAs from HC. HC_*trans*, mobile RNAs from HC. (B) Scatterplots of transcript abundance of mobile RNAs in HC versus CD samples. CD, mobile RNAs of CD origin. HC, mobile RNAs of HC origin. (C) Common set of transcripts between CD_*trans* (mobile RNAs from CD) RNAs and HI RNAs. (D) Common set of transcripts between HC_*trans* (mobile RNAs from HC) RNAs and HI RNAs. (E) Scatterplots of transcript abundance of mobile RNAs in CD versus HI samples. (F) Scatterplots of transcript abundance of mobile RNAs in HC versus HI samples.

Although mRNA transfer has been documented between stem parasitic plant in *Cuscuta* species and their hosts,^14^^,^^15^^,^^20^ the transfer mechanism remains poorly understood. It was hypothesized that mRNA transfer might be driven by specific selective determinants, such as abundance, size, featured motif, or specific modification of the mobile mRNAs.^5^^,^^15^^,^^21^^,^^22^ Therefore, we asked whether these characteristics of mobile mRNAs could influence the transfer events between root parasitic plant *C*. *deserticola* and host *H*. *ammodendron* or not. FPKM analyses showed that transcript abundance of the majority of mobile unigenes of *C*. *deserticola* origin was higher than that of non-mobile unigenes from same species. However, the transcript abundance of mobile mRNAs of *H*. *ammodendron* origin showed divergent patterns of comparable to higher FPKM values than those of non-mobile mRNAs from same species (Figure 3A). Moreover, mobile mRNAs of CD origin showed higher abundance in source than in destination tissue, where we speculate that the mobile mRNAs might be diluted or degraded after transfer event (Figure 3B). Compared with this, mobile mRNAs of HC origin showed no uniform correlation of transcript abundances between source and destination tissues (Figure 3B). These results demonstrated that mRNA transfer could be correlated with unigene abundance, but the mechanistic basis for such correlations remains obscure.

We assume that mobile mRNAs might be present in haustorium as they should translocate through parasitic interface. Most of the mobile unigenes could be found in haustoria unigene dataset, accounting for 99.21% (7,437/7,496) and 80.89% (1,917/2,370) of the transfer unigenes originated from *C*. *deserticola* and *H*. *ammodendron*, respectively (Figures 3C and 3D). This result indicated that root parasitic plant and its host exchanged their transcripts through haustorial junction, which serves as mechanical bridge between host and parasite and mediates the transfer event. Further evidence for haustorium-mediated selective mobility of unigenes came from plots of unigene abundance in haustorium versus abundance of the same unigenes in source *C*. *deserticola* and *H*. *ammodendron*, respectively. The plot of CD→HC mobile mRNAs showed that expression levels of most unigenes in the source CD sample were positively correlated with those in haustorium (Figure 3E). This result was similar to previous report about mobile mRNA between *Arabidopsis* and *Cuscuta* (Kim et al., 2014), indicating that most unigenes followed the same dynamics of movement. The plot of HC→CD mobile mRNAs showed a more-dispersed pattern of transcript abundances without obvious correlation between source HC and haustoria (Figure 3F). These results demonstrated that root parasitic plant *C*. *deserticola* and its host *H*. *ammodendron* could exchange their transcripts in a haustorium-mediated selective manner, but the dynamics of movement differed between two directions.

**Figure 4:**
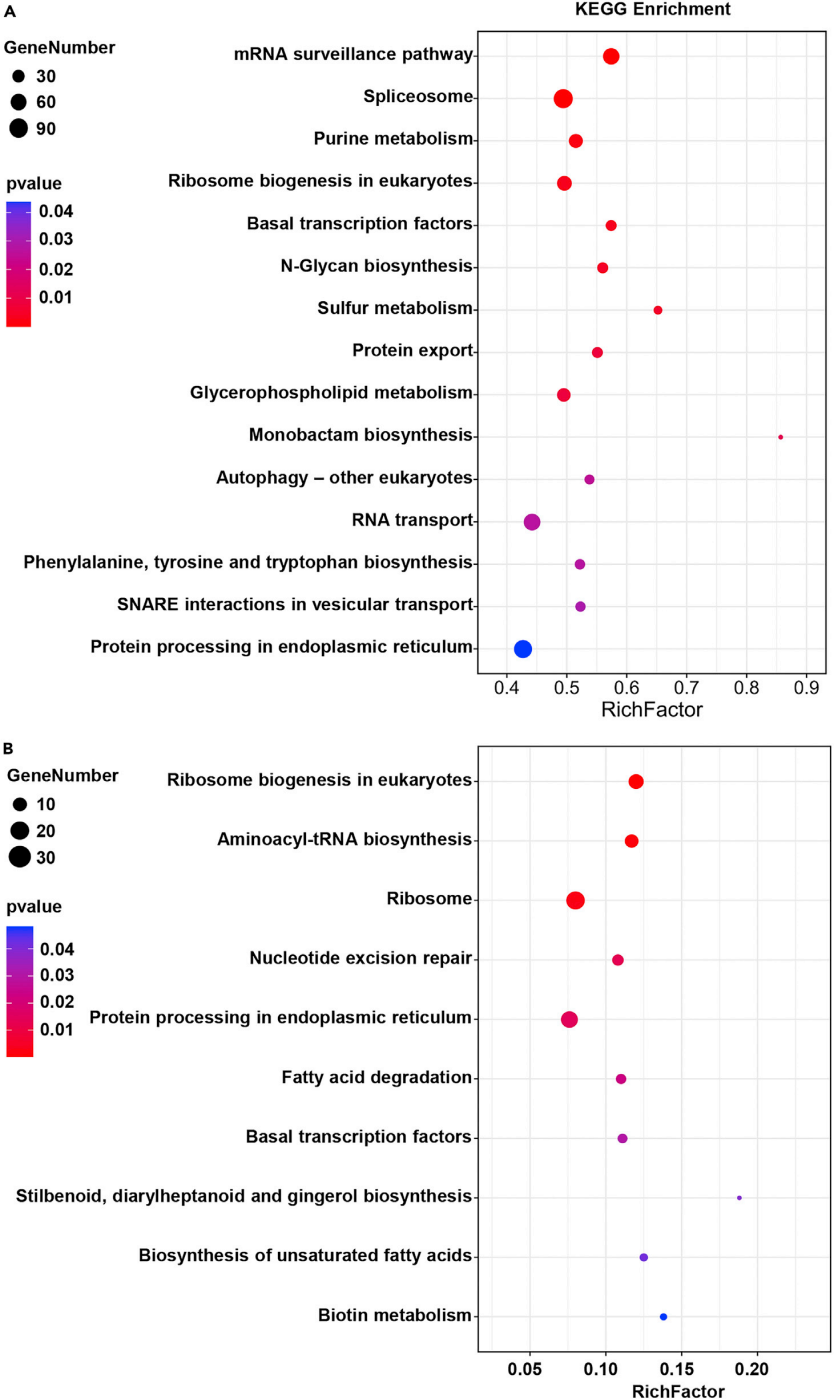
KEGG pathway analysis of mobile RNAs of CD origin (A) and HC origin (B) All the expressed genes of CD and HC were annotated in KEGG database, and were then used as background for enrichment analysis, respectively. The x axis represents the mobile RNA ratio to background in the pathway term. The circle size indicates the number of mobile RNAs that are associated with each significant pathway. The circle color indicates the significant level with the adjusted p value <0.05.

KEGG analyses showed the overall functions of the mobile mRNAs (Figures 4A and 4B). In the KEGG analysis, the terms related to RNA transport and metabolism, such as “RNA transport”, “spliceosome” and “mRNA surveillance pathway” were enriched in CD→HC mobile RNAs, whereas none of them were detected in HC→CD mobile RNAs. The enrichment of above pathways is consistent with the more frequent transfer of mRNA in CD→HC direction than that in the opposite direction (Figure 4A). “RNA transport” related mobile mRNAs in parasite *C*. *deserticola* might facilitate the large-scale transfer of mRNAs in the CD→HC direction. Furthermore, the presence of “spliceosome” and “mRNA surveillance pathway” related mobile RNAs from the parasite into host indicates that these mobile mRNAs might be involved in the maturation of parasite-derived mRNAs, or they may modify the mRNAs of host origin to influence the recipient plant’s physiology.^23^ In addition to “RNA transport”, “protein export” pathway was also enriched in CD→HC mobile RNAs (Figure 4A). More frequent transfer of proteins than RNAs was observed in the association of stem parasite dodder and its host *Arabidopsis* and soybean.^24^ The term “autophagy”, a process involved in programmed cell death (PCD) and immune response, was enriched in CD→HC mobile RNAs (Figure 4A). In HC→CD direction, metabolic pathway genes for protein biosynthesis are significantly enriched compared with CD→HC direction. The term “ribosome”, “protein processing in endoplasmic reticulum”, “ribosome biogenesis in eukaryotes” and “aminoacyl-tRNA biosynthesis” are all related to protein biosynthesis and related genes were highly represented in HC→CD mobile RNAs (Figure 4B).

### Experimental confirmation of selected mobile mRNAs

**Figure 5:**
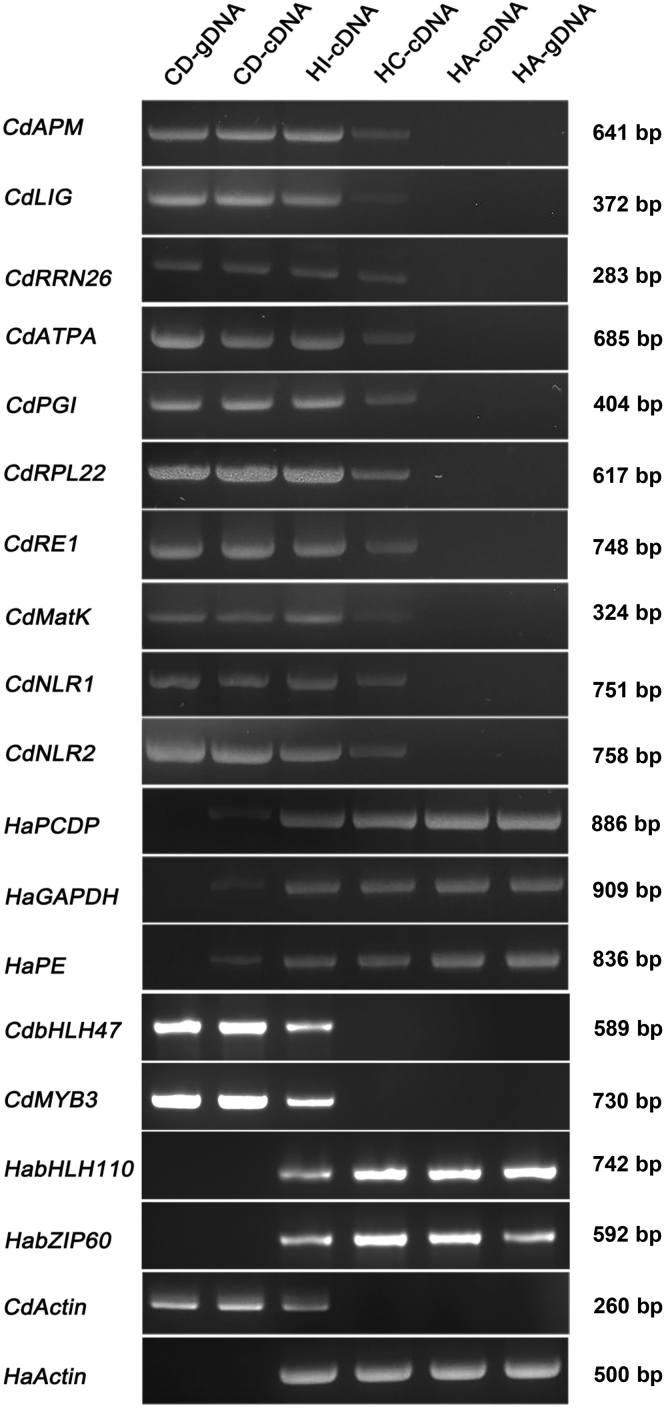
Confirmation of mobile mRNAs via PCR assay Total RNAs were isolated from HC, HA, CD and HI samples and were reverse transcribed into cDNAs. Genomic DNAs (gDNAs) were also isolated from HA and CD samples. Above cDNAs and gDNAs were used as the templates for PCR amplification of ten CD→HC mobile transcript genes and three HC→CD mobile transcript genes using gene specific primers. Both *HaActin* (*H*. *ammodendron Actin*) and *CdActin* (*C*. *deserticola Actin*) were amplified as internal control for host and parasite samples respectively. Gene names were shown on the left, and sample names are shown at the top panel. CD-gDNA, genomic DNA of *C*. *deserticola*. CD-cDNA, cDNA of *C*. *deserticola*. HI-cDNA, cDNA of haustorium. HC-cDNA, cDNA of *H*. *ammodendron* parasitized with *C*. *deserticola*. HA-gDNA, genomic DNA of *H*. *ammodendron*. HA-cDNA, cDNA of *H*. *ammodendron*. The experiments were performed three times and the representative pictures were shown. PCR procedure:98°C 5 min; 98°C 30s, 55°C 30s, 72°C 1 min, 35 cycles; 72°C 10 min.

We tried to test the independent transfer event but it was impossible to experimentally validate all of these mobile mRNAs. Therefore, we randomly selected some of the unigenes to verify the transfer event (Tables 1 and S3–S14). Polymerase chain reaction (PCR) assay was carried out to validate the mobile mRNAs and genomic DNAs (gDNAs) of both host and parasite were used as control templates. We reasoned that the PCR product could only be amplified from gDNA of source organism other than gDNA of destination organism for a specific mobile mRNA, as the mobile mRNA can only be transcribed from source gDNA but not from destination gDNA. To avoid the amplicon size differences between gDNA and cDNA, we used the gene fragments with no putative intron for PCR assay. We confirmed ten CD→HC and three HC→CD mobile mRNAs using this assay (Table 1 and Figures S3–S14). We could amplify HC→CD mobile sequences from HA gDNA, HA cDNA, HC cDNA and less abundantly from HI cDNA and CD cDNA while not from CD gDNA. Similarly, we could amplify CD→HC transfer sequence from CD gDNA, CD cDNA, and less abundantly from HI cDNA and HC cDNA while not from HA cDNA and HA gDNA (Figure 5). The presence of corresponding nucleic acids for mobile mRNAs in source gDNA but not in destination gDNA confirmed the transfer direction. Less abundant transcript level in destination than in source sample indicates that mobile mRNA is less represented in the recipient sample, which is consistent with the sequencing results (Figure 3B). As a negative control for this PCR experiment, we tested two HA non-mobile mRNAs (*HabHLH110*, *HabZIP60*) and two CD non-mobile mRNAs (*CdbHLH47*, *CdMYB3*), and we could not amplify them from CD cDNA or HC cDNA respectively (Figure 5). Above data indicated the reliability of the PCR results.

**Table 1:** Information of thirteen mobile and four non-mobile mRNAs for experimental validation

Gene Annotation	Transcriptome ID	Gene Symbol	Predicted ORF length (bp)	Transfer direction
Peptidase_M1 domain-containing protein	c121402_g2	CdAPM	510	CD→HC
DNA ligase	c114708_g2	CdLIG	2, 247	CD→HC
26S rRNA	c85065_g1	CdRRN26	NA	CD→HC
ATP synthase subunit A	c126123_g1	CdATPA	774	CD→HC
Glucose-6-phosphate isomerase	c119887_g2	CdPGI	747	CD→HC
50S ribosomal protein L22	c79535_g1	CdRPL22	507	CD→HC
Retrovirus-related pol polyprotein from transposon	c90862_g1	CdRE1	591	CD→HC
Maturase K	c55576_g1	CdMatK	1, 548	CD→HC
NB-ARC domain-containing protein	c137753_g2	CdNLR1	2, 247	CD→HC
NB-ARC domain-containing protein	c137753_g3	CdNLR2	1, 407	CD→HC
Programmed cell death protein	c139367_g1	HaPCDP	1, 959	HC→CD
Glyceraldehyde-3-phosphate dehydrogenase	c127689_g1	HaGAPDH	450	HC→CD
Pectinesterase	c127482_g1	HaPE	1, 068	HC→CD
Transcription factor bHLH110	c107962_g1	HabHLH110	1, 425	Non-transferred in HC
Transcription factor bZIP60	c108491_g1	HabZIP60	984	Non-transferred in HC
Transcription factor MYB3-like	c104027_g1	CdMYB3	897	Non-transferred in CD
Transcription factor bHLH47	c112633_g1	CdbHLH47	669	Non-transferred in CD

The ORF was predicted from unigenes by performing BLAST (E value = 1e^−5^) in the databases such as NR (NCBI non-redundant protein sequences) and SwissProt. ESTScan software was also used to confirm the annotation. Cd, *Cistanche deserticola*; Ha, *Haloxylon ammodendron*;NA, not applicable.

### Further confirmation and functional analysis of CD→HC mobile mRNAs

**Figure 6:**
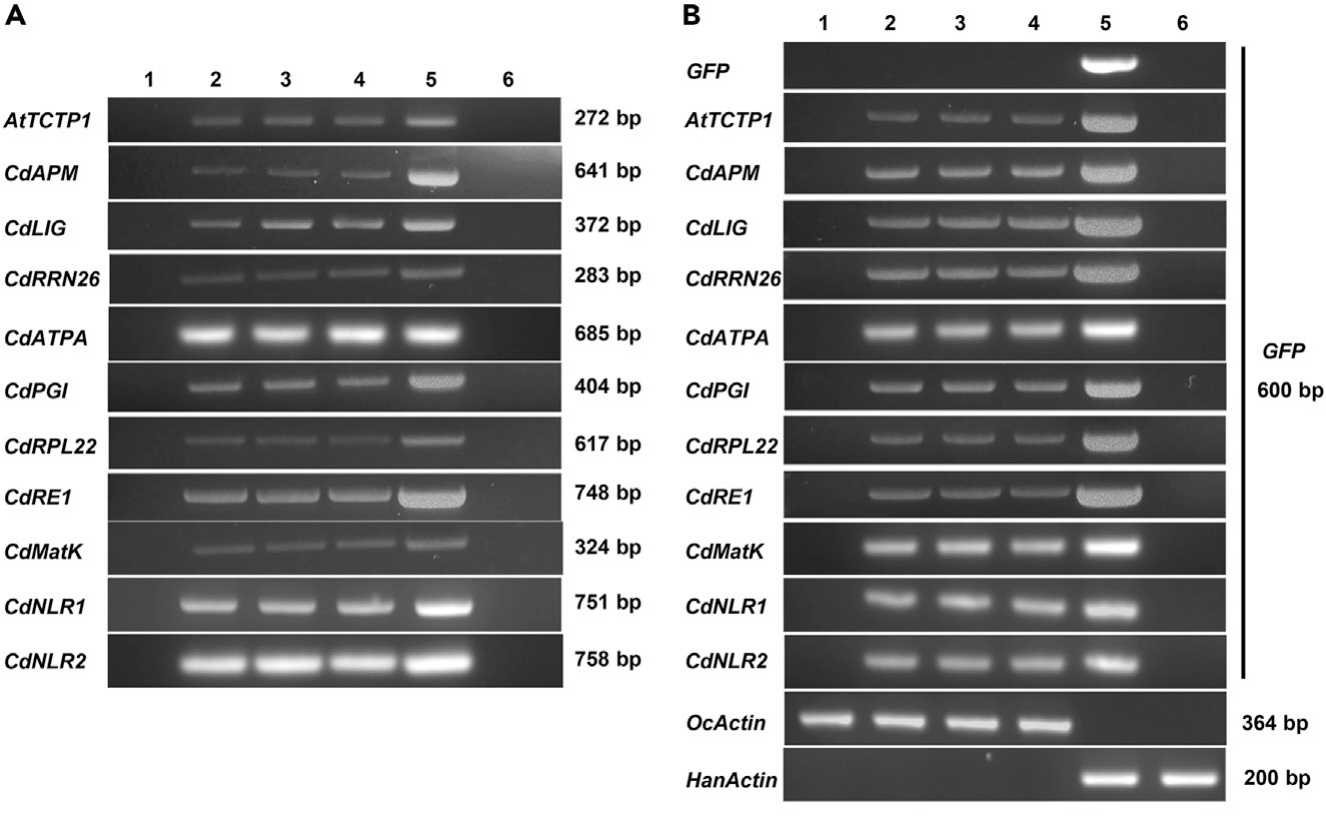
Confirmation of mobile mRNAs in sunflower-*O*. *cumana* transient expression assay Sunflower seed was soaked in GV3101 containing recombinant vectors carrying fused sequence of respective mobile transcript gene and *GFP*. *AtTCTP1* served as positive control and *GFP* alone served as negative control. Twenty-day-old seedlings were parasitized with 100 mg of *O*. *cumana* seeds per host. Total RNAs were isolated from the host sunflower and attached *O*. *cumana* samples, reverse transcribed into cDNAs, and were used as the templates for PCR amplification of transfer sequences. (A) PCR amplification with mobile transcript gene specific primers. Each panel represents the samples transformed with respective gene constructs and the gene name was shown on the left. (B) PCR amplification with *GFP* specific primers. The template was the same with (A) and sample name was shown on the right of each panel. Both *HanActin* (*Helianthus annuus Actin*) and *OcActin* (*Orobanche cumana Actin*) were amplified as internal control for host and parasite samples respectively. Lane 1, control *O*. *cumana* parasitized on non-transformed host; Lanes 2–4, three biological replicates of test *O*. *cumana* parasitized on transformed host; Lane 5, transformed host; Lane 6, non-transformed host. The experiments were performed three times and the representative pictures were shown.

Based on the above results, we aimed to further validate the transfer event by in planta overexpression and to characterize the function of the mobile mRNAs. We mainly studied CD→HC mobile mRNAs for validation as they were more broadly and confidently identified than HC→CD mobile RNAs. *H*. *ammodendron-C*. *deserticola* parasitization system is not applicable for this purpose as the transgenic approaches are not available for both the host and parasite. Therefore, we utilized sunflower-*O*. *cumana* parasitic system in which the latter belongs to *Orobanchae* species as the same with *C*. *deserticola*. First, we evaluated the phylogenetic relationship between root parasitic plants *C*. *deserticola* and *O*. *cumana*, as well as corresponding hosts *H*. *ammodendron* and *Helianthus annuus* via phylogenetic analyses using *rbcL* (RuBisCO large subunit) sequence. The evolutionary tree showed that *C*. *deserticola* had a close phylogenetic relationship with *O*. *cumana*, as was also the same for the two hosts. However, the stem parasitic plant *Cuscuta australis* had a far phylogenetic relationship with both parasitic plants and their hosts, which indicated the reliability of the heterologous system (Figure S15). Previously, we established a sunflower transient expression system in our lab by seed-soak agroinfiltration (SSA) method.^25^ Here we modified this SSA method by using pBI121 vector for overexpression of the genes for mobile mRNAs. Candidate gene was fused with GFP (green fluorescence protein) sequence and driven under constitutive CaMV 35S promoter for *in planta* expression in pBI121 vector. The recombinant constructs were transiently transformed into host sunflower via SSA method, and the parasite *O*. *cumana* was attached to the root of host seedling. Total RNA was isolated from both host and attached parasite, and the mobile transcript gene and *GFP* mRNA was detected with RT-PCR approach, in which host signal indicates the expression of candidate genes in the host and parasite signal denotes the successful transfer of mRNAs from host to parasite. An *A*. *thaliana* well-characterized mobile transcript gene *AtTCTP1* (Translationally Controlled Tumor Protein) was fused with *GFP* sequence to serve as positive control for transfer event and *GFP* alone served as negative control.^26^ Consistent amplification of these genes in transformed but not control host indicates the successful expression of them in the host plant (Figure 6A). PCR products were detectable in positive control *AtTCTP1*-*GFP* fusion group but not in negative control *GFP* group in the parasite (Figure 6). It indicates that *GFP* mRNA alone is not mobile, whereas fusion of mobile mRNA of *AtTCTP1* endows it the ability to move from host to parasite. RT-PCR analysis with both candidate gene specific and *GFP* specific primers confirmed the existence of the mRNAs for the candidate genes, although they were less abundantly accumulated in the parasite than in the host (Figure 6B). The results indicated the successful transfer of these candidate mobile mRNAs from host to parasite. We should note that the mechanism of CD→HA movement and sunflower→*O*. *cumana* movement of CD mRNA could be different, and that directionality is also of concern to experimental validation. However, the above data provide some evidence for the mobility of these mRNAs given the present technical shortage of transgenic approach for the study of host-parasite communication.

**Figure 7:**
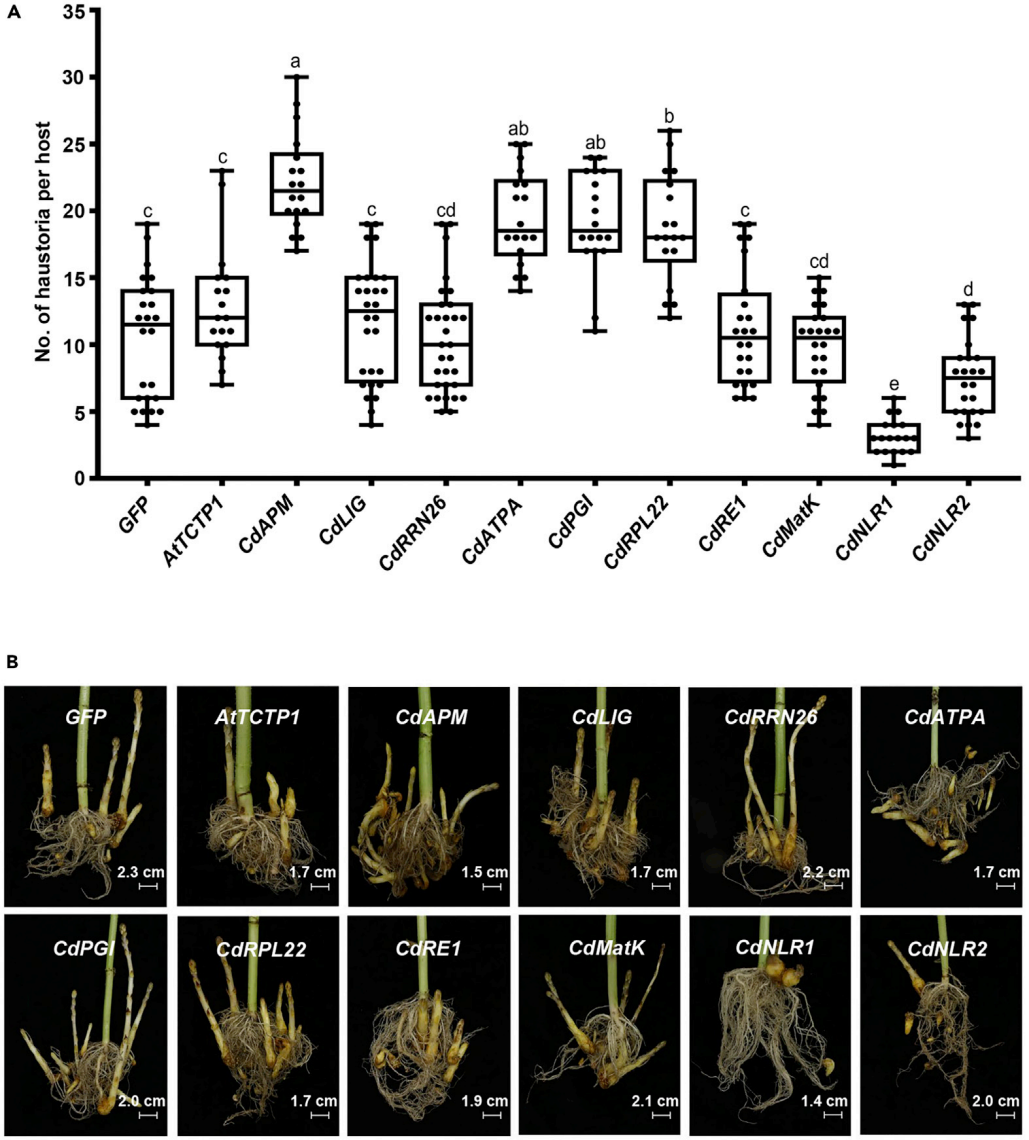
Phenotypic analyses of attached parasite on the expression of mobile mRNAs in sunflower-*O*. *cumana* transient expression assay Sunflower seed was soaked in GV3101 containing recombinant vectors carrying fused sequence of different mobile transcript gene and *GFP*. *AtTCTP1* served as positive control and *GFP* alone served as negative control. Twenty-day-old seedlings were parasitized with 100 mg of *O*. *cumana* seeds per host. The parasites were analyzed at 45 dpi. (A) Average number of haustoria formed on each host. Eighteen to thirty-one host plants were used for each group, and each dot indicates a biological replicate. Median number was indicated by short horizontal line and SE value was shown with vertical line. Statistic differences were analyzed by one-way ANOVA between different groups, and the different numbers indicate statistic difference with p*<0*.*05*. (B) Phenotypic observation of the parasites. Representative picture of parasites formed on the host root for each treatment was shown. Group name and scale bar were shown as inset for each figure.

We further investigated the role of mobile mRNAs on parasitic event. On the overexpression of above-verified mobile mRNAs, twenty-day-old sunflower seedlings were parasitized with *O*. *cumana*, and 45th day after parasitization *O*. *cumana* was removed from the soil for further analysis on haustorium development. As shown in Figure 7A, the number of haustorium (tubercle) formed on CdAPM, CdATPA, CdPGI and CdRPL22 groups increased by 78.1–110.5% whereas CdNLR1 and CdNLR2 groups decreased by 70.5% and 28.6% respectively, compared with control GFP group. There was no significant difference between five other treatment groups and the control GFP group. Representative parasites formed on sunflower root were shown in Figure 7B.

### *CdNLR1* and *CdNLR2* negatively regulate parasite development

**Figure 8:**
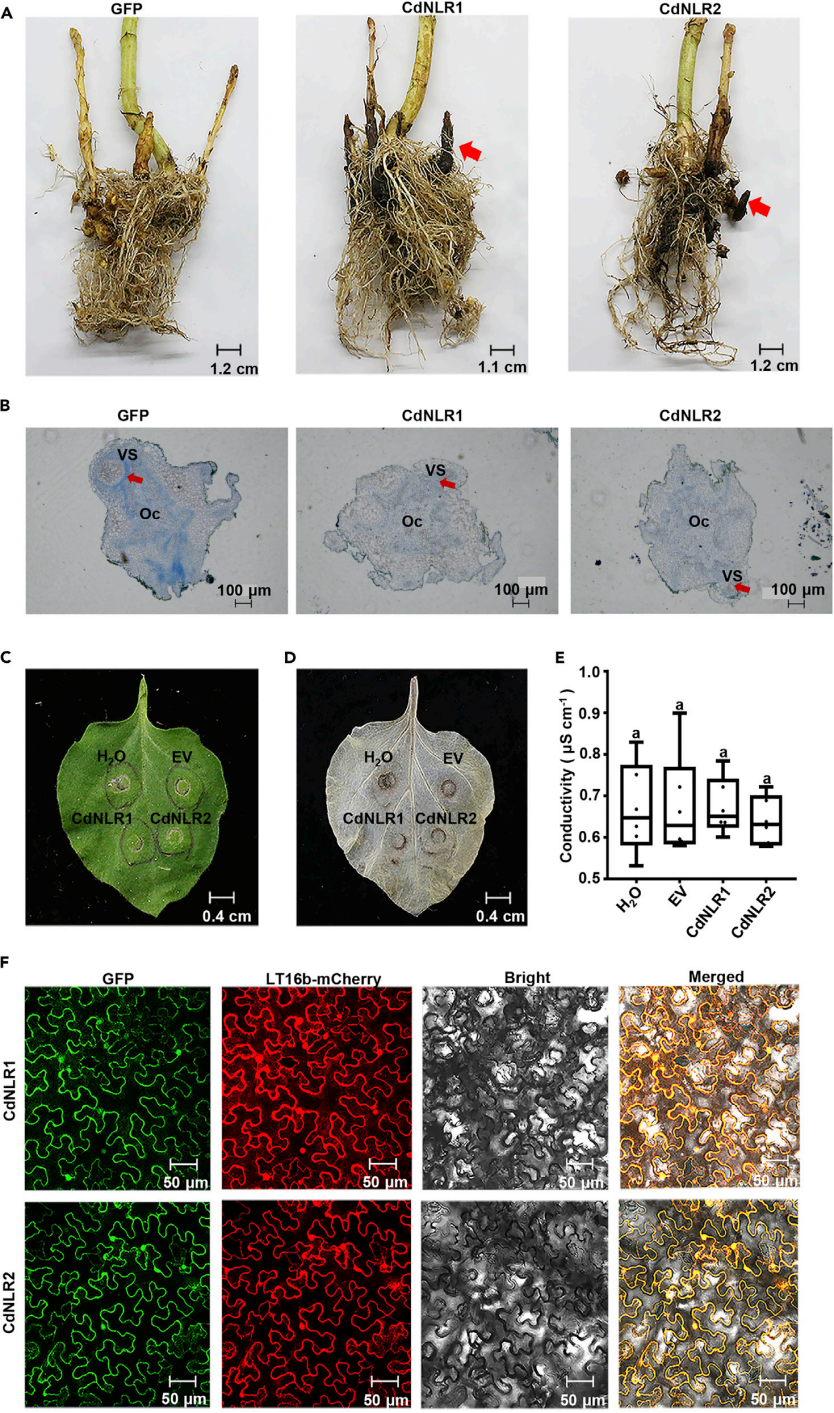
Functional confirmation of CdNLR1 and CdNLR2 (A) Necrotic phenotype of the tubercle attached on the host transformed with different constructs. Representative pictures for both healthy tubercles in control group and necrotic tubercles in CdNLR1 and CdNLR2 groups were shown. Red arrows denote the necrotic tubercles. Scale bar was shown. (B) Microscopic cross-sections for host root and parasite attachments. Oc, *O*. *cumana*; VS, vascular system of the sunflower root. Black arrow indicates the successful connection of *O*. *cumana* to the vascular system of the sunflower root. Scale bar was shown. (C–E) Transient expression assay in *N*. *benthamiana*. Plant leaves were infiltrated with *Agrobacterium tumefaciens* GV3101 inoculum (OD_600_ = 1.0) carrying different expression vectors and were visualized under natural light at 7 dpi (C). The same leaf was also photographed on DAB staining (D). Plant tissues were collected from infiltrated area, and electrolyte leakages was measured using a Horiba B-173 conductivity meter. The relative electrolyte leakage was expressed as the percentage of sample conductivity to total conductivity (E). Statistic differences were analyzed by one-way ANOVA between different groups. (F) Subcellular localization of CdNLR1 and CdNLR2. *N*. *benthamiana* leaves were infiltrated with *Agrobacterium tumefaciens* GV3101 containing recombinant pEarleyGate103-SL vector, and the subcellular localization of GFP-fused CdNLR1 and CdNLR2 was observed by confocal microscopy (Zeiss LSM710) under 635 nm red excitation light at 36 hpi. Plasma membrane marker protein LT16b fused with mCherry is shown.

*CdNLR1* and *CdNLR2* encoded resistance (R) proteins with putative NBS-LRR domain, respectively (Table 1). Given the importance of these two genes, we further confirmed the mobility of their mRNAs by detecting GFP signal in earlier haustorial development stage. On overexpression in the host sunflower, fusion of these two genes with *GFP* sequence results in clear GFP fluorescence signal in parasitic interface and young tubercle, indicating their ability for promoting transfer event (Figure S16). In later growth stage, overexpression of *CdNLR1* and *CdNLR2* caused visible tubercle necrosis which was not seen in control GFP group (Figure 8A). When the tubercle was observed at pre-elongation stage, compatible attachment was observed in the control GFP group, in which successful vasculature connection formed between host and parasite. In contrast, incompatible attachment was observed in *CdNLR1* and *CdNLR2* groups, where the haustoria did not successfully penetrate into host root and establish normal vascular continuity (Figure 8B). In consideration of the fact that R proteins generally caused hypersensitive response (HR) characterized by programmed cell death (PCD), we asked whether they induce HR in transient expression assay. When these genes were overexpressed on *Nicotiana benthamiana* leaves via agro-infiltration, overexpression of *CdNLR1* and *CdNLR2* did not cause any visible HR on the infected area (Figure 8C). Furthermore, DAB staining and measurement of cell death index also indicate that *CdNLR1* and *CdNLR2* expression did not induce HR in leaf tissue (Figures 8D and 8E). Subcellular localization analysis by GFP-fused expression of CdNLR1 and CdNLR2 in an *N*. *benthamiana* transient expression system showed that they localized to the cell membrane (Figure 8F). We speculated that CdNLR1 and CdNLR2 caused HR only in root but not in leaf tissue.

**Figure 9:**
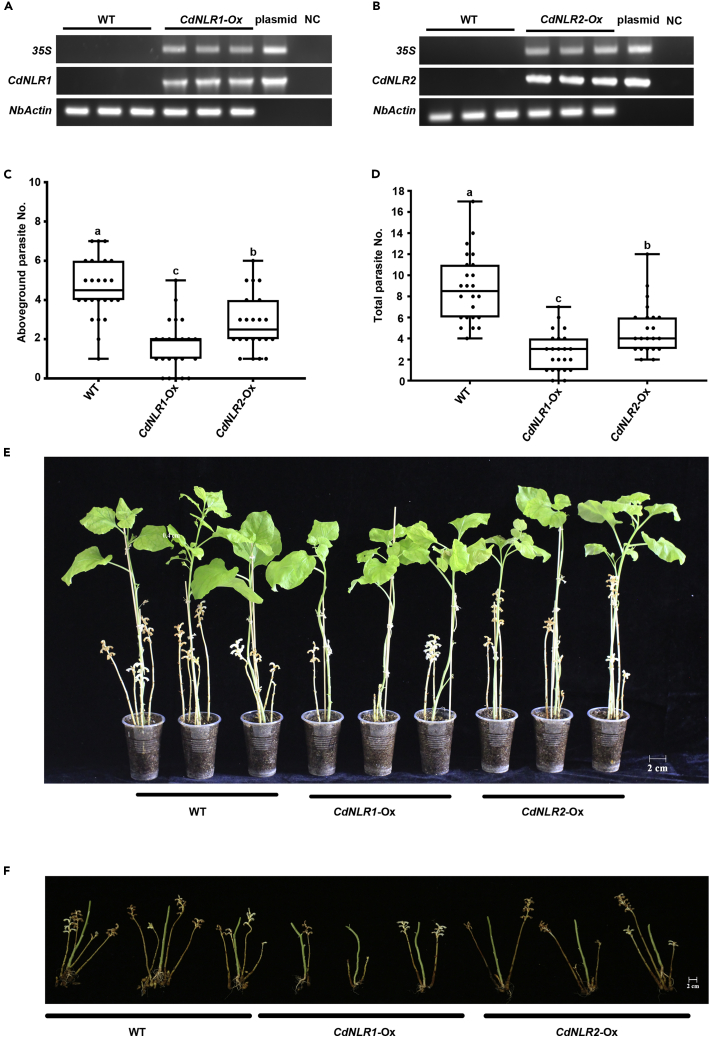
Development of transgenic plants and phenotypic analyses (A) and (B) PCR detection of transgenic plants. Upper lane: Genomic PCR confirmation of transgenic plants with 35S promoter specific primers. Middle lane: RT-PCR confirmation of transgenic plants with *CdNLR1* or *CdNLR2* specific primers. Lower lane: *NbActin* served as internal control for RT-PCR experiment. WT, wild type plant. *CdNLR1*-Ox, *CdNLR1* overexpression plant. *CdNLR2*-Ox, *CdNLR2* overexpression plant. Plasmid, recombinant plsmid for *CdNLR1*-Ox or *CdNLR2*-Ox construct. NC, negative control water for PCR assay. (C) The number of aboveground parasites formed on each individual. Six-leaf stage *N*. *benthamiana* seedling was parasitized with 100 mg of *O*. *cumana* seeds per host. At 50 days after inoculation, the number of emerged parasites was calculated. (D) The number of total parasites formed on each individual. At 50 days post inoculation, the soil was removed and the number of parasites formed on host root was calculated. (E) and (F) Representative picture of parasites for (C) and (D).

To further investigate the role of *CdNLR1* and *CdNLR2*, we made transgenic *N*. *benthamiana* overexpressing these two genes underCaMV 35S promoter, namely *CdNLR1*-Ox and *CdNLR2-*Ox plants respectively. Both genomic DNA PCR with 35S promoter specific primers (upper lanes) and RT-PCR with *CdNLR1* or *CdNLR2* specific primers (middle lanes) confirmed the corresponding transgenic lines (Figures 9A and 9B). As *O*. *cumana* could parasitize on Solanaceae species including *N*. *benthamiana*, six-leaf stage transgenic plant seedlings were parasitized with *O*. *cumana*, and 50 days after parasitization *O*. *cumana* was removed from the soil for further analysis on parasite development. The number of emerging above-ground parasites on *CdNLR1*-Ox and *CdNLR2-*Ox plants decreased by 64% and 38%, and the underground parasites decreased by 69% and 44% respectively, compared with those on WT (wild type) plant (Figures 9C–9F). We should also note that overexpression of *CdNLR1* and *CdNLR2* did not cause any abnormal phenotype, especially HR, in both leaf and root tissue, as is consistent with the data in Figures 8D and 8E. Taken together, this transgenic result confirmed the negative role of *CdNLR1* and *CdNLR2* in parasite development.

## Discussion

Cross-species RNA movement was reported between host plant and the interacting organisms. Artificial RNA transfer from host to pathogen was intensely reported for many pathogenic organisms such as nematodes, insects and fungi in HIGS (host-induced gene silencing) experiment.^27^ Recently, host mediated RNAi toward pathogen gene was also verified in parasitic plants.^11^^,^^28^^,^^29^ The above literature demonstrated the effective movement of ectopically expressed siRNA, a hallmark of RNAi, in host-pathogen interactions. Compared with this, natural RNA transfer event was rarely reported and the functional relevance of them were even less investigated. If any, such studies were mainly reported for small RNAs such as nat-siRNA (natural siRNA) and miRNA.^30^ Accordingly, it was shown recently that sRNAs moved via vascular structures between host and pathogens including bacteria, fungi and parasitic plants.^12^^,^^13^^,^^31^^,^^32^ Several pathogens deliver sRNAs into host cells to inhibit the immunity. For example, a set of 22 nt miRNAs could be delivered from the parasitic plant *Cuscuta campestris* to the hosts *A*. *thaliana* and *N*. *benthamiana* to suppress expression of target genes *TIR1*, *AFB2*, *AFB3*, *BIK1* and *SEOR1* to establish successful parasitism.^12^ Furthermore, large-scale sRNA exchanges were also reported for *Arabidopsis*-*Cuscuta* parasite system.^33^

Compared with sRNAs, it is difficult to investigate mRNA transfer due to the technical hurdles for distinction of long transcript sequence between source and destination organisms.^2^^,^^34^ In this regard, parasitic plants offer a good option for the study of mobile mRNAs as they are evolutionarily distant from their host, and therefore sufficient sequence variation allow them to be distinguished between host and parasite. In early attempt, mRNA transfer was firstly confirmed for limited genes from host to parasite unidirection.^14^ Large-scale identification of mobile mRNAs was achieved via microarray analysis and sequencing technology for model hosts *Arabidopsis*, tomato and well-studied parasite dodder.^14^^,^^15^ It is difficult to distinguish the mRNA between source and destination tissues, especially for non-model species which generally lack genomic data^35^ (Westwood & Kim, 2017). However, now this issue is beginning to be overcome with the increases of sequencing depth and the improvement in bioinformatic approaches. Using multiple sequence alignment and comparative analysis, we identified around ten thousand mobile mRNAs between woody plant *H*. *ammodendron* and root-attached *C*. *deserticola*. In *Arabidopsis*-*cuscuta* connection, mobile mRNAs of 9,518 and 8,655 were originated from *Arabidopsis* and *cuscuta* respectively, accounting for 45% and 24% of the transcripts from source organism.^15^ However, in the same report, only 347 (1.6%) and 288 (1.1%) mobile unigenes were identified in tomato-*cuscuta* parasitic system.^15^ More recently, Liu et al. reported that 172 (0.06%) and 1,416 (0.28%) mobile mRNAs were exchanged between *Arabidopsis* and dodder, while mobile mRNAs were only 64 (0.12%) and 708 (0.19%) in soybean-dodder connection.^24^ We report here that 2,370 (3.38%) and 7,496 (9.66%) unigenes moved between *H*. *ammodendron* and *C*. *deserticola*, respectively. Therefore, the scope and transfer specificity of RNA trafficking varied between different parasite systems. The present study represents a first report of mRNA transfer for root parasite and it differs from stem parasitic plant dodder. More confluent transfer of mRNA in CD→HA direction than the opposite direction might be contributable to species differences, however, it needs further investigation to be confirmed.

The most challenging issue for mobile RNA study is experimental verification of transfer event and functional characterization of mobile RNAs. Transcriptome sequencing is in depth and large scale, and confirmation of transfer mRNA could be facilitated by multiple sequence alignment as the host and parasite are distantly related to each other.^15^^,^^19^ Here, we showed that sequence comparison with the orthologs from closely related species aided to identify the origin of mobile mRNA, whereas ruling out the sequence from distantly related species which represent the destination organism. Although the biological function of mobile mRNA is well studied for variety of genes within an organism, their implications in plant-parasite connections are poorly understood.^2^ This is mainly hampered by the lack of effective research tool for mobile mRNA, and is especially true for the system in which transgenic approach is lacking. To date, functional verification of mobile mRNAs was only achieved in model parasite system such as *Arabidopsis*-dodder and soybean-dodder in which transgenic methods are available, and for well-studied genes such as herbicide phosphinothricin acetyltransferase (*PAT*)^36^ and florigen *FT* gene.^37^ Here, we used sunflower-*O*. *cumana* system to facilitate both experimental validation and functional study of mobile mRNAs and confirmed their roles in parasite development. Overexpression of four *C*. *deserticola* genes *CdAPM*, *CdATPA*, *CdPGI* and *CdRPL22* was shown to expedite haustorium attachment, indicating that these four genes possessed promotive roles in parasite attachment and haustorium development. Although less studied, the involvement of above genes for haustorium development was marginally reported. For example, several V-type ATP synthase/ATPase proteins were enriched in extrahaustorial space of powdery mildew-infected barley leaves to enhance disease susceptibility. *FgGPI*, a Glucose-6-phosphate isomerase gene which is involved in ATP biosynthesis and carbon source utilization, is required for hyphal growth and fungal development of *Fusarium graminearum*.^38^ Taken together, our study demonstrates the effectiveness of sunflower-*O*. *cumana* parasitic system for the functional validation of *C*. *deserticola* genes and this strategy could also be useful for other root parasites.

It should be noted that *C*. *deserticola* is a specialist parasite that has only one reported host *H*. *ammodendron*, which is a sand-grown shrub of limited biotope in nature.^17^ From an evolutionary aspect, excessive parasitism of *C*. *deserticola* would cause growth failure of host and renders both host and parasite to unfavorable growth situation. Therefore, it is reasonable to speculate that this narrow host-parasite relationship in natural ecosystem might evolve an equilibrium mechanism by which the parasite on a given host is restricted to a limited number for successful fulfillment of life cycle, instead of killing the host for their own death. Of interest, overexpression of *CdNLR1* and *CdNLR2*, two *C*. *deserticola NBS-LRR* genes encoding putative R proteins, caused dramatic decrease in tubercle development. Therefore, transfer of PCD-inducing mobile mRNAs for *CdNLR2* and *CdNLR3* might determine individual parasite destiny and prevents their over-parasitism on a host. Although the hypersensitive response was seldom reported for host-parasitic plant interactions compared with other host-pathogen system, it might also contribute to host immunity against parasitic plants. In stem parasite *Cuscuta spp*. and tomato association, a hypersensitive-like response (HLR) was reported to occur for incompatibility and to be mediated via plant hormone salicylic acid (SA).^39^^,^^40^ For example, Cowpea (*Vigna unguiculata*) cultivar B301 encodes VuPOB1, a host BTB-BACK domain-containing ubiquitin E3 ligase homolog, to cause hypersensitive response at the site of parasite attachment on the invasion of races SG4 and SG3 of the root parasitic weed *Striga gesnerioides*.^41^

### Limitations of the study

In this study, we identified around ten thousand mobile mRNAs between a woody host and a root parasite. However, it remains to be determined whether all these mobile mRNAs have functional implications in host-parasite interactions. Furthermore, lack of genetic manipulation for such non-model species hinders functional verification of the mobile mRNAs in original plant species; rather, we verified their roles in heterologous system. Therefore, the present study provides some evidence for cross-species mRNA transfer event whereas the individual mRNA role await further study. The functional correlation of transfer event and phenotypic outcome in recipient species will be unveiled in future study.

## STAR★Methods

### Key resources table

**Table:** 

REAGENT or RESOURCE	SOURCE	IDENTIFIER
**Bacterial and virus strains**		

*Agrobaterium tumefaciens* strain GV3101	Weidi	AC1001S
Trans1-T1 Phage Resistant Chemically Competent Cell	Transgen	Code#CD501-02

**Critical commercial assays**		

Clontech SMARTer PCR cDNA Synthesis Kit	Takara	Cat# 634925

**Deposited data**		

Raw sequencing reads for *Haloxylon ammodendron* and *Cistanche deserticola*	This paper	NCBI GenBank BioProject: PRJNA768605
Illumina reads of transcriptomes from *Triphysaria versicolor*, *Striga hermonthica* and *Phelipanche aegyptiaca*	Westwood et al.^42^	Parasitic Plant Genome Project (http://ppgp.huck.psu.edu/)
Genome assemblies from 32 angiosperm Species	NCBI, Phytozome v13, etc.	See details in Table S12
Illumina reads of transcriptomes from *Haloxylon ammodendron*	Long et al.; Gao et al.^43^^,^^44^	NCBI GenBank: GSE63970 and GSE93684

**Experimental models: Organisms/strains**		

*Haloxylon ammodendron* and *Cistanche deserticola*	Wild populations collected from Dengkou, Bayinnor, China	N/A
*Nicotiana benthamiana*	This paper	N/A
*Helianthus annuus* and *Orobanche cumana*	Wild populations collected from Wuchuan, Hohhot, China	N/A

**Oligonucleotides**		

For all oligonucleotides used for cloning and genotyping	This paper	See details in Table S1

**Recombinant DNA**		

pBI121-GFP	This paper	N/A
pBI121-GFP-AtTCTP1/CdAPM/CdLIG et al.	This paper	N/A
pEarleyGate103-CdNLR1/CdNLR2	This paper	N/A
pMD1-LTI6b-mCherry	Cutler et al.;^45^ modified in this paper	N/A

**Software and algorithms**		

Trimmomatic v0.33	Bolger et al.^46^	https://github.com/timflutre/trimmomatic
Trinity v2.3.2	Grabherr et al.^47^	https://github.com/trinityrnaseq/trinityrnaseq
BLAST v2.2.28	Camacho et al.^48^	https://blast.ncbi.nlm.nih.gov/Blast.cgi
RSEM v1.2.15	Li et al.^49^	http://deweylab.github.io/RSEM/
ESTScan v3.0.3	Iseli et al.^50^	http://estscan.sourceforge.net/
SMRTlink v5.1	Chin et al.^51^	https://www.pacb.com/support/software-downloads/
LoRDEC	Salmela et al.^52^	http://www.atgc-montpellier.fr/lordec/
OrthoFinder v2.5.2	Emms & Kelly^53^	https://github.com/davidemms/OrthoFinder/releases
PlantRegMap	Tian et al.^54^	http://plantregmap.gao-lab.org/

### Resource availability

#### Lead contact

Further information and requests for resources and reagents should be directed to and will be fulfilled by the lead contact, Hada Wuriyanghan (nmhadawu77@imu.edu.cn).

#### Materials availability

This study did not generate new unique reagents.

### Experimental model and subject details

#### Plant materials

*Cistanche deserticola* (Y. C. Ma) and *Haloxylon ammodendron* (C. A. Mey) were grown in a desert field at Dengkou, Bayinnor (40°45' N, 106°24' E) in China. The region has a typical continental arid climate with an average annual precipitation of 182 mm, about 70% of which occurs from July to September. Mean annual temperature of this region is 8.5°C, and mean temperature of the coldest (January) and the hottest (July) months are −15.7°C and 30°C, respectively. *C. deserticola* seed was evenly spread around and on top of the root of host *H. ammodendron* seedlings. At 80 days post inoculation, the parasite achieves the elongation stage and were used for transcriptome sequencing.

Sunflower cultivar (HN3638) were grown in growth chambers, and the *Orobanche cumana* (race G)-sunflower parasitic system was established in our lab.^25^ Briefly, *O. cumana* race G seeds were collected in bulk from a highly infested field in Hohhot (China; latitude, 41.09289° N, longitude, 111.45785° E) during autumn 2018. The same sunflower cultivar (HN3638) that was heavily parasitized with *O. cumana* in the field was used for inoculation as it is highly susceptible to the *O. cumana* material we used here. Sunflower seed was sown in soil in pots and grown for 20 d to reach a height of ∼8 cm. The soil was carefully removed to expose host root, and surface-sterilized *O. cumana* seeds (100 mg per host) were evenly spread around and on top of the root tissue.

#### Bacterial growth

For the Agrobacterium-mediated transient expression, the cells of *Agrobacterium tumefaciens* strain GV3101 harboring the appropriate plasmids were cultured in LB broth containing appropriate antibiotics for vector selection, and the broth cultures were grown at 28°C and 200 rpm. See Method details for details.

### Method details

#### Sample collection, library construction and transcriptome sequencing

The fresh succulent stems of *C. deserticola* (abbreviated as CD hereafter, and the same for other samples) 1 cm away from the haustorial junction was collected. At the same time, the roots of non-parasitized *H. ammodendron* (HA), the roots of CD-infested *H. ammodendron* (HC), and the haustorial interface (HI) were collected. Tissues were carefully harvested to avoid any chance of mixture and were rinsed for 10 min in 70% ethanol. Total RNA was extracted by Trizol reagent from individual plant tissue. RNA concentration was analyzed by NanoDrop2000 spectrophotometry and the RNA integrity value (RIN) was determined by Bioanalyzer 2100 system. Three biological replicates were performed for CD, HA and HC, and one replicate was used for HI. Ten μg of the total RNA, pooled from five individuals, was used for library construction, and RNA-seq was performed according to the methods described in our previous publications.^55^

#### Transcriptome assembly and annotation

Raw reads in FASTQ input files were processed through in-house Perl script (https://www.perlscriptsjavascripts.com/perl/ba_faq/index.html). Adapter sequence, ploy-N reads and other low-quality reads were removed from raw data to obtain clean reads. Q20, GC content and sequence duplication level of the clean data were calculated. *De novo* assemblies of clean reads were achieved via Trinity software.^47^ First, combined sequences were obtained by hybrid assembly of the reads from all ten libraries containing CD, HA, HC and HI samples. Second, the reads from six libraries containing both HA and HC were assembled to obtain *H. ammodendron* transcripts (designated as HAC hereafter). Third, the reads from three CD libraries were assembled to obtain *C. deserticola* transcripts (designated as Cis hereafter).^47^ Redundant transcripts were filtered from above assemblies to obtain the unigenes. BLASTX and ESTScan (version 3.0.3) was used to remove the sequences of non-coding regions and to predict putative proteins with open reading frames (ORFs).^48^^,^^50^ Non-redundant unigenes were utilized to perform BLAST (E value = 1e^−10^) search and unigene function was annotated by the databases such as Nr (NCBI nonredundant protein sequences), Pfam (Protein family), KOG/COG (euKaryotic Ortholog Groups/Clusters of Orthologous Groups of proteins), Swiss-Prot (A manually annotated and reviewed protein sequence database), KO (KEGG Ortholog) and GO (Gene Ontology).

#### Full-length transcriptome sequencing and processing

Full-length transcriptomes of *C. deserticola* and non-parasitized *H. ammodendron* were also obtained by third-generation sequencing. The isoform sequencing library was prepared according to the Iso-Seq^TM^ protocol as described by Pacific Biosciences. Briefly, 0.5 μg of poly(A) RNA was reverse transcribed into cDNA using Clontech SMARTer^TM^ cDNA Synthesis Kit (Takara, Japan). BluePippin system was used for size selection of cDNAs, and SMRTbell^TM^ libraries were constructed via PacBio Template Prep Kit (Pacific Biosciences, USA). The libraries were sequenced on a PacBio Sequel sequencer by Novogene company (Beijing, China). Sequence data were processed via SMRTlink 5.1 software.^51^ Circular consensus sequence (CCS) was generated from subread BAM files. CCS.BAM files were retrieved and classified into full-length and non-full-length reads via isoform-level clustering (ICE). Additional nucleotide errors in consensus reads were corrected using the Illumina RNA-seq data with the LoRDEC software.^52^ Any redundancy in consensus reads was removed by CD-hit to obtain final transcripts of *C. deserticola* and non-parasitized *H. ammodendron* (abbreviated as CD_FL and HA_FL hereafter) for the subsequent analysis.^56^

#### Orthogroup clustering and gene loss analysis

Orthogroups were created with OrthoFinder (v2.5.2) and were used the investigation of gene loss and construction of phylogenetic tree.^53^ To this aim, the predicted proteins of *C. deserticola*, *H. ammodendron*, three different parasitic plants in Orobanchaceae,^42^ and complete proteomes of thirty-two sequenced flowering plants were used (Table S14). All major representative flowering plant clades were selected for this analysis. Generated orthogroups in thirty-seven species were list in Data S1. The orthogroups present in at least three of the five asterid species, *Coffea canephora*, *Helianthus annuus*, *Mimulus guttatus*, *Sesamum indicum* and *Solanum tuberosum* were identified and were considered to be conserved across the asterids. We subsequently used them for assessing gene loss in *C. deserticola*. As a comparison, we also examined gene loss in four hemiparasitic plant species (*Phtheirospermum japonicum*, *Triphysaria versicolor*, *Striga asiatica, and Striga hermonthica*), and two holoparasitic plant species (*Cuscuta australis* and *Phelipancheaegyptiaca*). For functional analysis of missing orthogroups from *C. deserticola* and six additional parasitic plants, we performed GO analysis using PlantRegMap to determine enriched terms.^54^ Missing orthogroups from *M. guttatus* were used as the foreground gene set with *p*-value threshold of 0.05.

#### Personalized analysis of sequencing data and identification of mobile mRNAs

Unigene expression levels were evaluated by RSEM (RNA-Seq by Expectation Maximization) analyses.^49^ Transcriptome reads of CD, HC, HA and HI were mapped to Combined unigenes to calculate the expression level of unigenes in above four samples, and unigenes with FPKM (Fragments Per Kilobase per Million) ≥ 0.3 in at least one replicate (three replicates in CD, HC and HA, and one replicate in Hau) were used for downstream analysis. Principal component analysis (PCA) was performed to confirm the similarity between samples. To avoid possible mistakes during hybrid assembly, all assigned unigenes were separately filtered via dual BLASTN (E value = 1e^−10^) against four local libraries (unigenes of Cis and HAC, full-length non-redundant transcripts of CD and HA), and the union sets were retained. Venn diagram analyses between CD, HC and HA was performed to identify common and differed unigenes among these samples. The candidate transfer sequences were blasted to a series of sequence datasets to classify the sequence origin. Custom tailored Perl programming (www.perl.org) and Python programming (www.python.org) were used to facilitate this analysis. As *H. ammodendron* and *C. deserticola* belong to different families, Chenopodiaceae and Orobanchaceae, respectively, we hypothesized that the available sequence data from these two families would help to identify the source organism by homology searching. Therefore, the candidate transfer unigenes were further separated with dual BLASTN (E value = 1e^−10^) against a collection of public available datasets from Orobanchaceae and Chenopodiaceae species. For Chenopodiaceae, we used the following datasets: *H. ammodendron*, next-generation transcriptome GSE63970 and GSE93684,^43^^,^^44^ full-length non-reduntant transcriptome in this manuscript; EST and mRNA sequences downloaded from NCBI database. For Orobanchaceae, we used the following databases: transcriptome of *Triphysaria versicolor, Striga hermonthica* and *Phelipancheaegyptiaca*,^57^ EST and mRNA sequences downloaded from NCBI database. The putative CD→HC transfer mRNAs were compared with Orobanchaceae datasets and the low similarity sequences were removed. Similarly, the putative HC→CD transfer mRNAs were compared with Chenopodiaceae datasets, and the low similarity sequences were removed. At the same time, the highly conserved orthologs between HC and CD which also had high similarity with both Chenopodiaceae and Orobanchaceae were filtered out.

#### PCR confirmation of mobile mRNAs

Total RNA was isolated by Trizol reagent and genomic DNA contamination was removed by DNase treatment. RNA quality and concentration were detected by NanoDrop2000 spectrophotometer. GoScript reverse transcription system (Promega, A5000) was used for cDNA synthesis. Genomic DNA was isolated and RNA contamination was removed by RNase treatment. Both cDNA and genomic DNA were used as the templates for PCR amplification. The primers used were shown in Table S1. PCR reaction was performed, and the amplicons were separated on 1% agarose gel, with DNA marker run on the same gel for the estimation of band size. Representative amplicons from genomic DNA and cDNA were sequenced to verify the sequences.

#### pBI121 vector construction, transformation and parasite infestation

The ORF sequences for mobile mRNA genes were amplified from CD cDNA with the gene specific primers (Table S1). The amplicons were first inserted into cloning vector and were verified by sequencing. The sequences were then subcloned into pBI121 vector. GFP (Green fluorescent protein) sequence was amplified from pJL24 vector and C-terminally fused to these genes. Recombinant pBI121 vectors were transformed into competent cell of *Agrobaterium tumefaciens* strain GV3101 respectively, and cultured cells (OD_600_ = 0.5) were suspended in the inoculation solution (10 mM MES, 10 mM MgCl_2_, 200 μM AS, 5% sucrose). Inoculation to sunflower seed was carried out according to the seed soak agroinfiltration method established in our lab.^25^ Twenty daysold seedlings were parasitized with 100 mg of sterilized *O. cumana* seeds which were evenly spread around and on top of the root of sunflower seedlings. The seedlings were cultured at the growth chamber, and the parasites were taken from the soil for RT-qPCR assay, histological characterization and phenotypic observation. For subcellular localization, ORF sequence for full length *CdNLR1* and *CdNLR2* was amplified and was ligated into pEarleyGate103-SL vector. The recombinant vector was transformed to *Agrobacterium tumefaciens* GV3101, infiltrated into *N. benthamiana* leaves, and GFP signal (excitation at 488 nm) was visualized at 36–48 hpi. DAPI-stained nuclei were imaged using excitation at 405 nm and fluorescence detection at 425–493 nm.

### Quantification and statistical analysis

All statistical analysis in this paper were analyzed by one way ANOVA between different groups using GraphPad Prism 8.0.2, and the different numbers indicate statistic difference with *P*<0.05.

## Data Availability

The Illumina and PacBio raw sequence data for *C. deserticola* and *H. ammodendron* reported in this study have been deposited in the NCBI Sequence Read Archive (SRA) underBioproject PRJNA768605 (https://www.ncbi.nlm.nih.gov/bioproject/PRJNA768605). Other existing, publicly available data used in this paper can be seen in method details. This paper does not report original code. Data reported in this paper and any additional information required to reanalyze the data will be provided from the lead contact upon request. The data that support the finding of this study are available in the supplementary material of this article.

## Supplemental information

Document S1. Figures S1–S16 and Tables S2–S15

Table S1. Primers used in this study, related to STAR Methods

Data S1. Orthogroups in *Cistanche deserticola*, *Haloxylon ammodendron* and other thirty-five plant species, related to STAR Methods

Data S2. Missing orthogroups (A) and genes (B) in *Cistanche deserticola* and other six parasitic plants, related to Figure 2 Cis, *Cistanche deserticola*; Cus, *Cuscuta australis*; Str, *Striga asiatica*; StHe, *Striga hermonthica*; TrVe, *Triphysaria versicolor*; OrAe, *Phelipanche aegyptiaca*; Pht, *Phtheirospermum japonicum*.

Data S3. Enriched gene ontology (GO) terms for missing orthogroups in *Cistanche deserticola* (A), *Cuscuta australis* (B), *Phelipanche aegyptiaca* (C), *Phtheirospermum japonicum* (D), *Striga hermonthica* (E), *Striga asiatica* (F) and *Triphysaria versicolor* (G), related to Figure 2
