# Supplementary material for: Large-scale mRNA transfer between Haloxylon ammodendron (Chenopodiaceae) and herbaceous root holoparasite Cistanche deserticola (Orobanchaceae)
Source: iScience. 2022 Dec 27;26(1):105880. doi: 10.1016/j.isci.2022.105880 (PMC9852350; doi:10.1016/j.isci.2022.105880)
Supplement: Document S1. Figures S1–S16 and Tables S2–S15 [file mmc1.pdf]

iScience, Volume *26*

Supplemental information

Large-scale mRNA transfer between *Haloxylon ammodendron* (Chenopodiaceae) and herbaceous root holoparasite *Cistanche deserticola* (Orobanchaceae)

Yanyan Fan, Qiqi Zhao, Huimin Duan, Shuxin Bi, Xiaomin Hao, Rui Xu, Runyao Bai, Ruonan Yu, Wenting Lu, Tiejun Bao, and Hada Wuriyanghan

Figure S1. Principal component analysis of the transcriptome data in *Haloxylon ammodendron* (HA, HC), *Cistanche deserticola* (CD) and the haustorial interface (HI), related to Figure 1. PCA was performed based on the FPKM (fragments per kilobase of transcript per million mapped reads) values of all the detected genes. One sample was shown in HI, and three replicate samples were shown in HA, HC and CD, respectively.

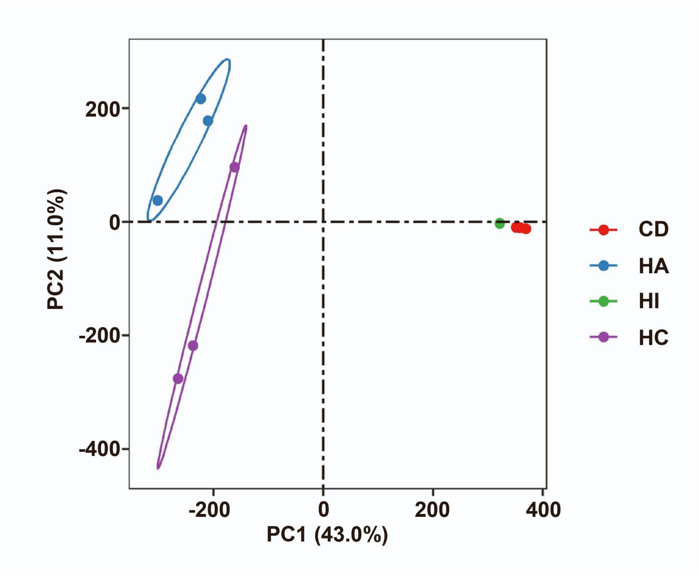

Figure S2. Venn of missing orthogroups between *Cistanche deserticola* and three other sequenced parasitic plants, including *Cuscuta australis*, *Striga asiatica* and *Phtheirospermum japonicum*, related to Figure 2.

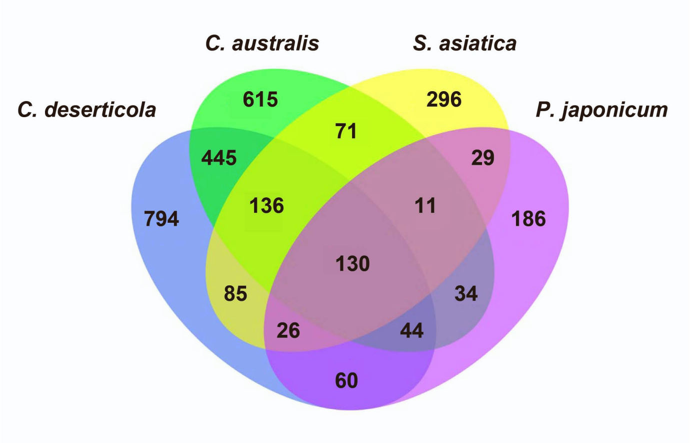

Figure S3-S14. Phylogenetic analysis of thirteen selected genes, related to Table 1. The genes of interest in this study were shown in red. The protein sequences were subject to multiple sequence alignment using ClustalW2. MEGA-X and iTOL website (https://itol.embl.de/) was used to build the phylogenetic tree via the Neighbor-Joining method, and bootstrap method was used to verify the quality of tree building with 1,000 times of inspection. Cd, *Cistanche deserticola*; Ha, *Haloxylon ammodendron*.

Figure S3. Phylogenetic analysis of *APM* genes, related to Table 1. Hs, *Hibiscus syriacus*; So, *Spinacia oleracea*; Mn, *Morus notabilis*; Rm, *Rhizophora mucronata*; La, *Lupinus angustifolius*; Dz, *Durio zibethinus*; Pt, *Populus trichocarpa*; Cca, *Cuscuta campestris*; St, *Solanum tuberosum*; Hu, *Herrania umbratical*; Tc, *Theobroma cacao*; Gb, *Gossypium barbadense*; Cc, *Corchorus capsularis*; Vv, *Vitis vinifera*; Ns, *Nyssa sinensis*.

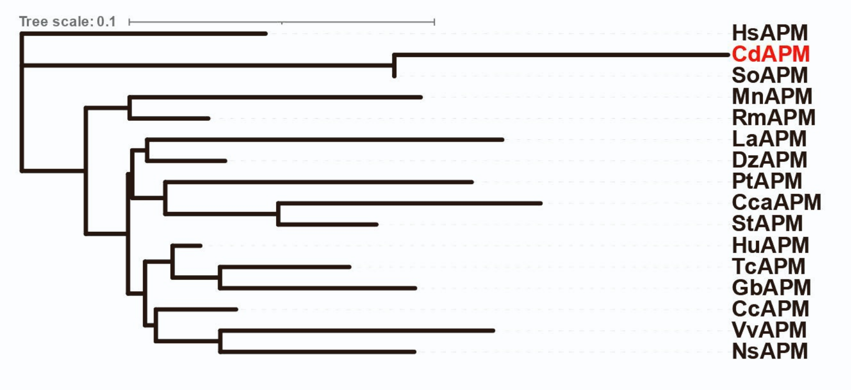

Figure S4. Phylogenetic analysis of *LIG* genes, related to Table 1.

Nn, *Nelumbo nucifera*; So, *Spinacia oleracea*; Di, *Davidia involucrate*; Ac, *Actinidia chinensis*; Han, *Helianthus annuus*; Car, *Coffea arabica*; Si, *Sesamum indicum*; Na, *Nicotiana attenuate*; Cch, *Capsicum chinense*; Sl, *Solanum lycopersicum*; Eg, *Eucalyptus grandis*; Pg, *Punica granatum*; Gs, *Glycine soja*; Mt, *Medicago truncatula*; Ca, *Cicer arietinum*; Zj, *Ziziphus jujuba*; Mn, *Morus notabilis*; Jr, *Juglans regia*; Ql, *Quercus lobata*; Dz, *Durio zibethinus*; Gt, *Gossypium tomentosum*; Sv, *Salix viminalis*; Pa, *Populus alba*; Ccl, *Citrus clementina*; Cf, *Cephalotus follicularis*.

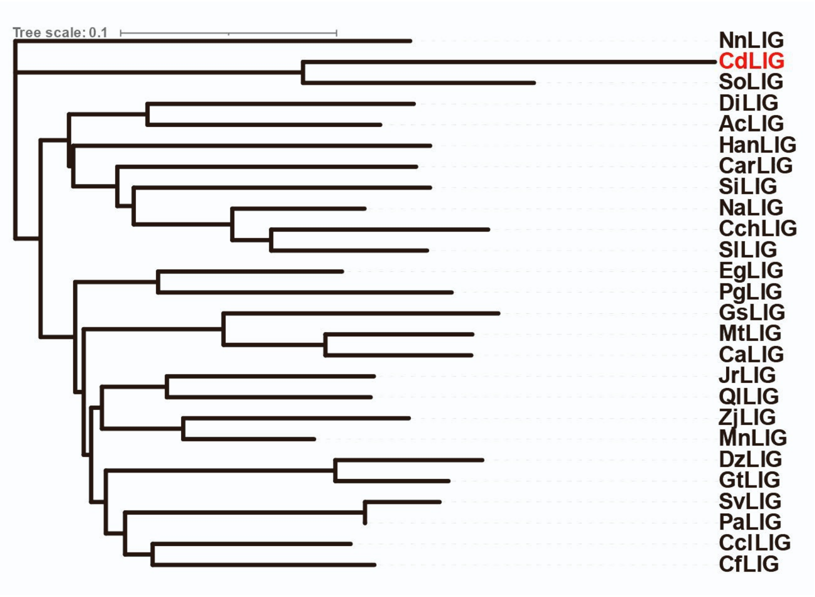

Figure S5. Phylogenetic analysis of *RRN26* genes, related to Table 1. Cs, *Camellia sinensis*; Sh, *Scyphiphora hydrophyllacea*; Car, *Coffea arabica*; Pp, *Plocama pendula*; Mg, *Mimulus guttatus*; Pv, *Pyrostegia venusta*.

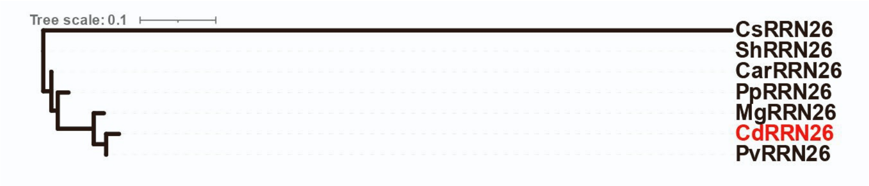

Figure S6. Phylogenetic analysis of *ATPA* genes, related to Table 1. Ss, *Salvia splendens*; Cm, *Cinnamomum micranthum*; Np, *Neobartsia pedicularoides*; Bs, *Boechera stricta*; Tg, *Turritis glabra*; Bv, *Beta vulgaris*; Cl, *Citrullus lanatus*; Cp, *Cucurbita pepo*; Cb, *Capsella bursa*; Ppi, *Pongamia pinnata*; Lj, *Lotus japonicus*; Cma, *Clerodendrum mandarinorum*; Ev, *Eruca vesicaria*; Rs, *Raphanus sativus*; Bj, *Brassica juncea*; Sa, *Striga asiatica*; Hn, *Hyoscyamus niger*; Han, *Helianthus annuus*; Aa, *Arabis alpina*; At, *Arabidopsis thaliana*.

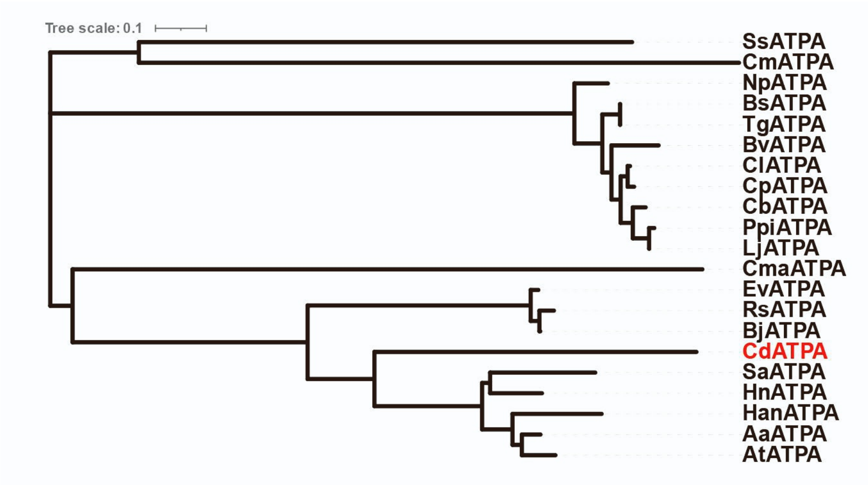

Figure S7. Phylogenetic analysis of *PGI* genes, related to Table 1. Sb, *Sorghum bicolor*; Zm, *Zea mays*; Pan, *Phaseolus angularis*; Tp, *Trifolium pratense*; Vv, *Vitis vinifera*; Aco, *Ananas comosus*; Aan, *Artemisia annua*; Ccl, *Citrus clementina*; Gr, *Gossypium raimondii*; Han, *Helianthus annuus*; Atr, *Amborella trichopoda*; Cf, *Cephalotus follicularis*; Fs, *Fagus sylvatica*; Eg, *Eucalyptus grandis*; Pg, *Punica granatum*; Tw, *Tripterygium wilfordii*; Jc, *Jatropha curcas*; Sv, *Salix viminalis*; Nt, *Nicotiana tabacum*; Sc, *Solanum chacoense*; Os, *Opuntia streptacantha*; Cq, *Chenopodium quinoa*; So, *Spinacia oleracea*; Ccaj, *Cajanus cajan*; Gm, *Glycine max*; Ad, *Arachis duranensis*; Ccan, *Coffea canephora*; Csa, *Cannabis sativa*; Pang, *Parasponia andersonii*; To, *Trema orientale*.

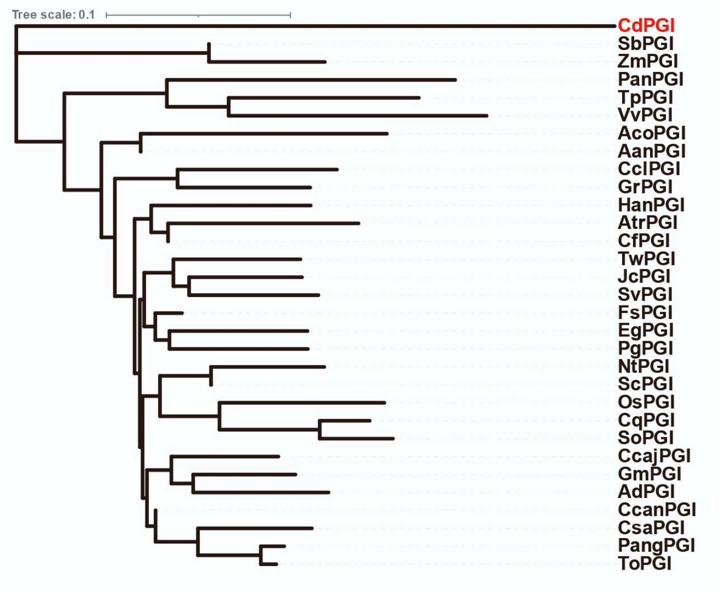

Figure S8. Phylogenetic analysis of *RPL22* genes, related to Table 1. Ai, *Aeginetia indica*; El, *Erythranthe lutea*; Pl, *Phryma leptostachya*; Rh, *Rehmannia henryi*; Pk, *Paulownia kawakamii*; Ba, *Buddleja alternifolia*; Vc, *Verbascum chinense*; Aca, *Aphyllon californicum*; Pi, *Pedicularis ishidoyana*; Tv, *Triphysaria versicolor*; Cpa, *Castilleja paramensis*; Sam, *Schwalbea americana*; Pj, *Phtheirospermum japonicum*; Bsw, *Brandisia swinglei*; Ls, *Lathraea squamaria*; Er, *Euphrasia regelii*; Ni, *Neobartsia inaequalis*.

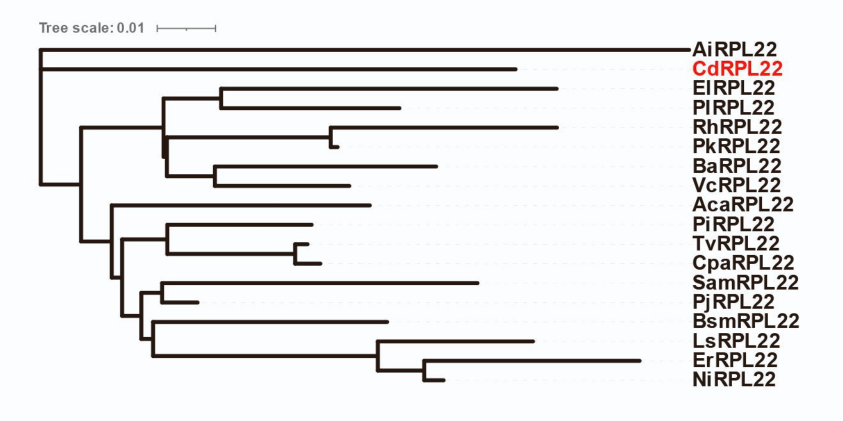

Figure S9. Phylogenetic analysis of *RE1* genes, related to Table 1. At, *Arabidopsis thaliana*; Tci, *Tanacetum cinerariifolium*; Mp, *Marchantia polymorpha*; Aan, *Artemisia annua*; Han, *Helianthus annuus*; Sl, *Solanum lycopersicum*; Cme, *Cucumis melo*; Mn, *Morus notabilis*; Nt, *Nicotiana tabacum*; Csi, *Citrus sinensis*; Ccaj, *Cajanus cajan*; Dc, *Dendrobium catenatum*; Hs, *Hibiscus syriacus*; Vv, *Vitis vinifera*; Dh, *Dorcoceras hygrometricum*; Tp, *Trifolium pratense*; Gs, *Glycine soja*;.

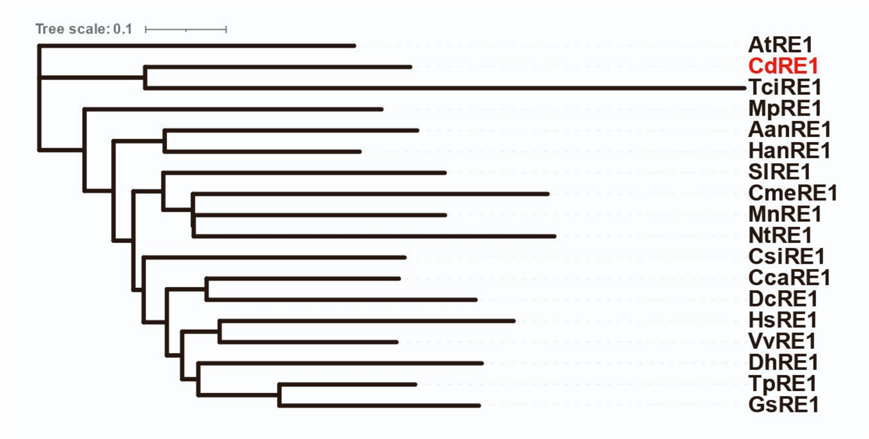

Figure S10. Phylogenetic analysis of *MatK* genes, related to Table 1. Sla, *Sopubia lanata*; Acap, *Alectra capensis*; Xb, *Xizangia bartschioides*; Phth, *Phtheirospermum* sp. 2 WBY-2017; Pn, *Pterygiella nigrescens*; Ma, *Mimulus alatus*; Rsi, *Radermachera sinica*; Si, *Sesamum indicum*; Tpa, *Tabebuia palustris*; Hi, *Handroanthus impetiginosus*.

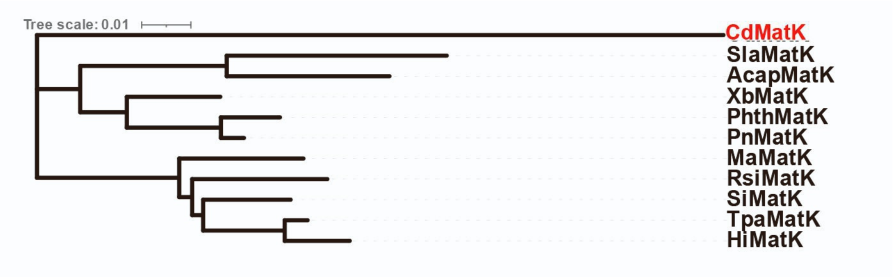

Figure S11. Phylogenetic analysis of *NLR* genes, related to Table 1. Hi, *Handroanthus impetiginosus*; Si, *Sesamum indicum*; Cau, *Cuscuta australis*; Sa, *Striga asiatica*; Egu, *Erythranthe guttata*.

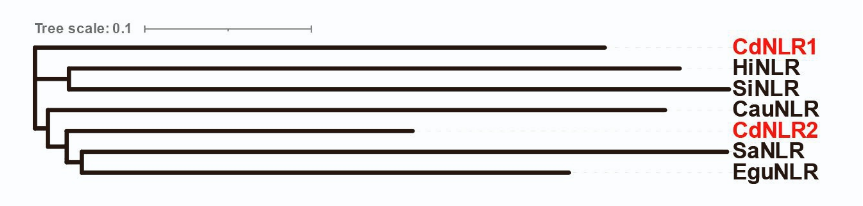

Figure S12. Phylogenetic analysis of *PCDP* genes, related to Table 1. Vv, *Vitis vinifera*; Car, *Coffea arabica*; Ac, *Actinidia chinensis*; Di, *Davidia involucrate*; Gs, *Glycine soja*; Ccaj, *Cajanus cajan*; Vu, *Vigna unguiculata*; Pa, *Populus alba*; Co, *Corchorus olitorius*; Ga, *Gossypium austral*; Tc, *Theobroma cacao*.

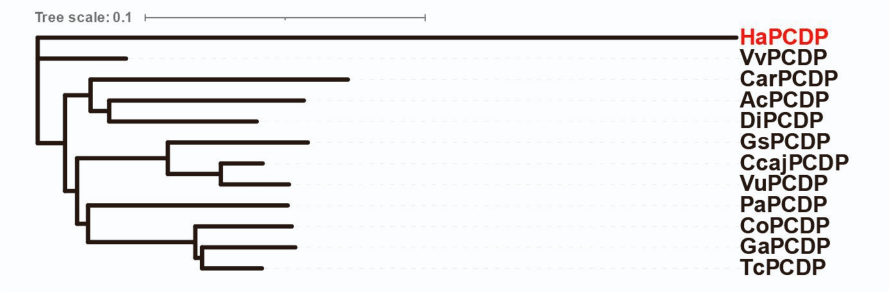

Figure S13. Phylogenetic analysis of *GAPDH* genes, related to Table 1.

Zm, *Zea mays*; Cba, *Capsicum baccatum*; Gmu, *Gossypium mustelinum*; Kb, *Koelreuteria bipinnata*; Cm, *Cinnamomum micranthum*; Car, *Coffea arabica*; Ccl, *Citrus clementina*; Ad, *Arachis duranensis*; Bv, *Beta vulgaris*; So, *Spinacia oleracea*; Aa, *Arabis alpina*; Bo, *Brassica oleracea*; Pg, *Punica granatum*; Ag, *Apium graveolens*; Dca, *Daucus carota*; As, *Angelica sinensis*.

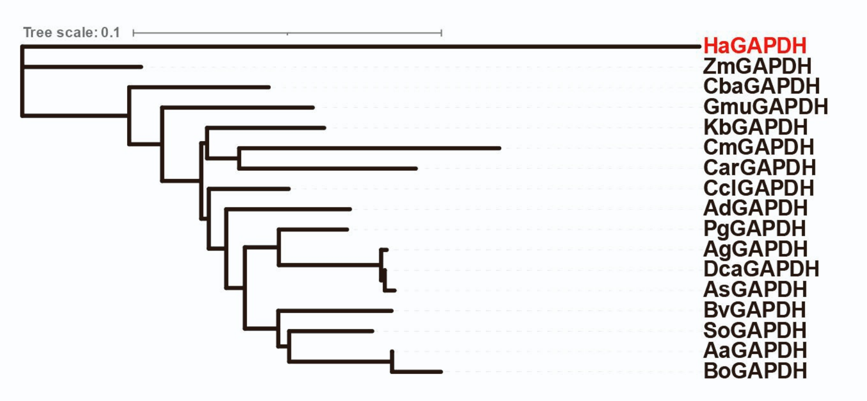

Figure S14. Phylogenetic analysis of *PE* genes, related to Table 1. Rco, *Ricinus communis*; Rc, *Rosa chinensis*; Ay, *Acer yangbiense*; Dh, *Dorcoceras hygrometricum*; Pt, *Populus trichocarpa*; Mn, *Morus notabilis*; Mm, *Mikania micrantha*; Eg, *Eucalyptus grandis*; Zj, *Ziziphus jujuba*; Vv, *Vitis vinifera*; Di, *Davidia involucrate*; Ns, *Nyssa sinensis*.

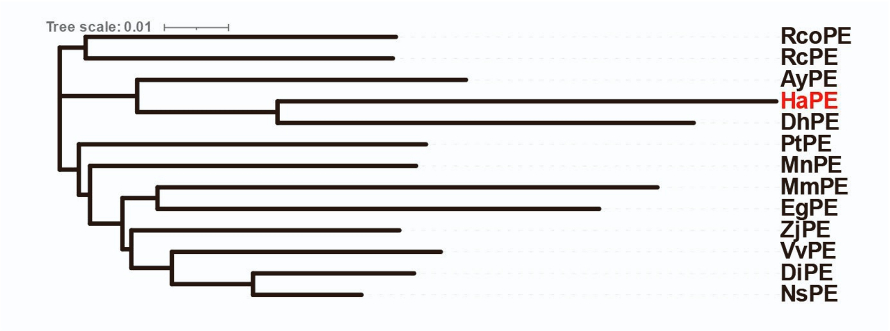

Figures S15. Phylogenetic tree of parasitic plants and hosts based on *rbcL*, related to STAR Methods. *Cuscuta australis* served as the outgroup.

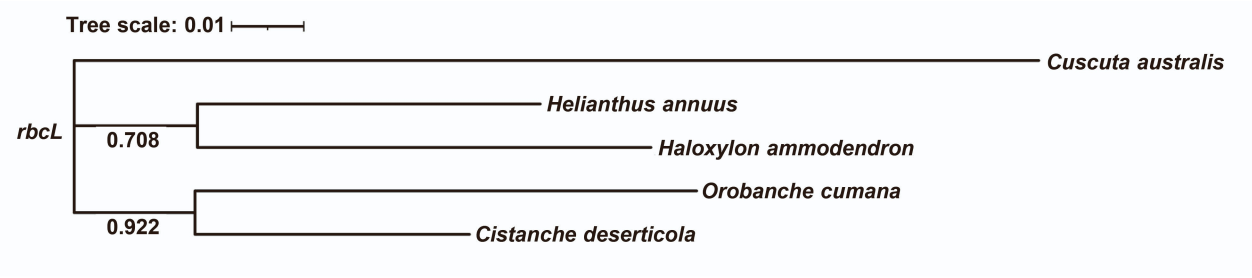

Figures S16. Detection of GFP fluorescence in sunflower-*O. cumana* parasitic system, related to Figure 8. Sunflower seed was soaked in GV3101 containing recombinant vectors carrying the sequence of CdNLR1 or CdNLR2 fused with GFP. GFP fluorescence was observed after haustoria formation. GFP severed as the negative control. Han, sunflower; Oc, *O. cumana*.

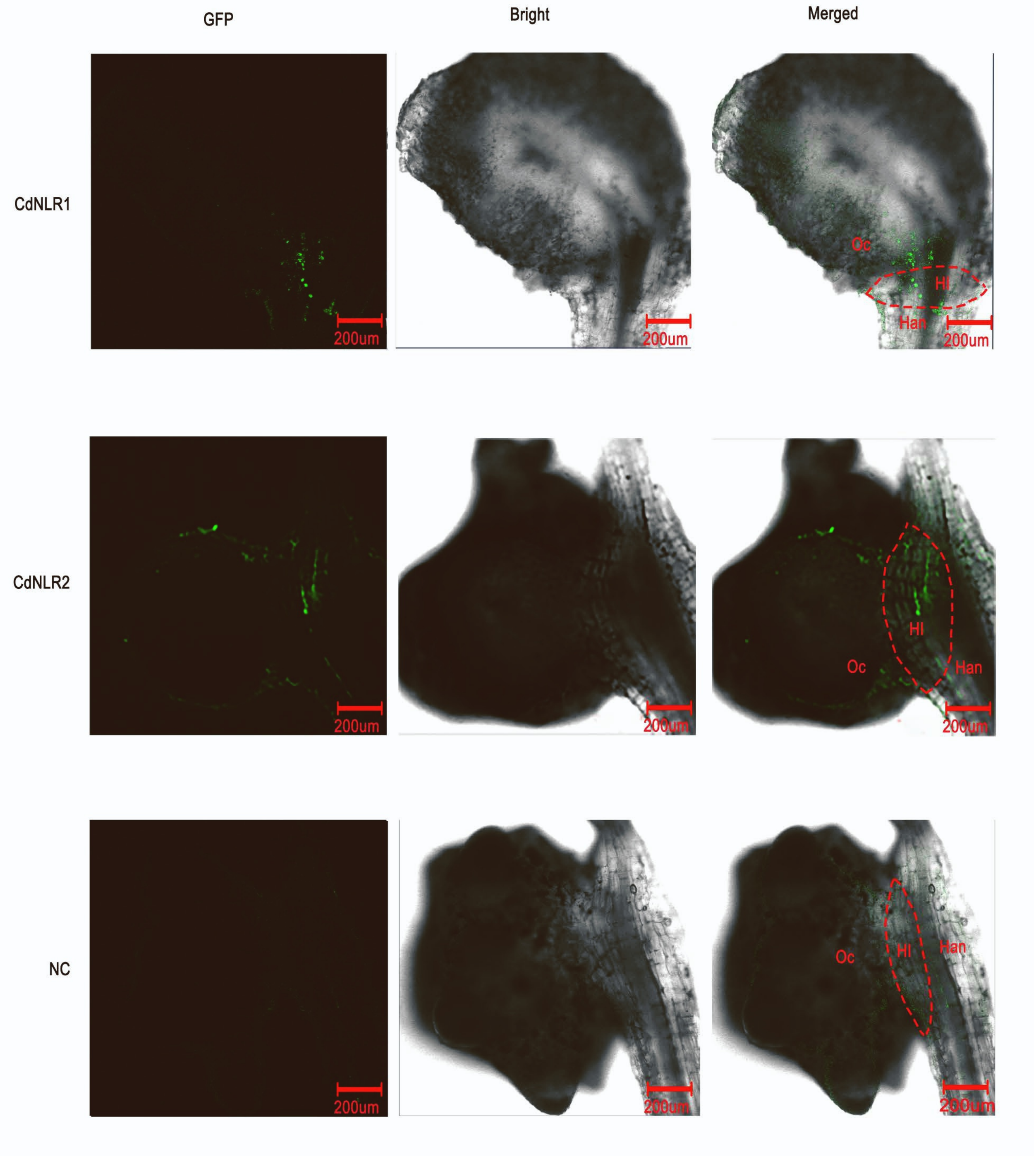

Table S2. Basic information of transcriptome data, related to Figure 1. HA, *H. ammodendron*; HC, *H. ammodendron* parasited with *C. deserticola*; CD, *C. deserticola*; Hau, haustorium of *C. deserticola*.

Sample	Raw Reads	Clean reads	Clean bases	Q20 (%)	GC (%)
**HA1**	78390576	75985174	9.50G	95.85	42.66
**HA2**	71884264	69655872	8.71G	95.90	42.68
**HA3**	72139852	69818110	8.73G	95.86	42.00
**CD1**	68825230	66106018	8.26G	95.28	45.64
**CD2**	72534882	69831450	8.73G	95.39	45.55
**CD3**	71575766	68885312	8.61G	95.36	45.35
**HC1**	83144142	80308854	10.04G	95.69	42.89
**HC2**	67366140	65263948	8.16G	95.61	43.08
**HC3**	95487526	92156022	11.52G	95.72	42.33
**Hau**	68453586	65372180	8.17G	95.10	46.04
**Total**	749,801,964	723,382,940	90.43G	NA	NA

Table S3. Primary transcriptome assembly statistics for all species used in this study, related to Figure 1. Three different assembly strategies were used in this study, and the longest transcript was identified as non-redundant unigene. HAC, unigenes combined assembly for HA and HC samples. Cis, unigenes assembly for CD samples. Combined, unigenes combined assembly for all ten samples.

Sample	200-500 bp	500-1k bp	1k-2k bp	>2k bp	Mean Length	N50	Total
**HAC**	128,089	36,246	19,758	10,627	631	961	194,720
**Cis**	73,661	17,297	10,885	5,909	615	982	107,752
**Combined**	139,751	39,827	25,996	17,325	710	1,250	222,899

Table S4. Coding sequence (CDS) prediction of Combined unigenes, related to Figure 1. The unigenes were screened against the NCBI non-redundant protein database and Swissprot using BLASTX (E value = $1e^{-5}$), and ESTScan was then used for de novo prediction for non-blasted unigenes. The longest predicted CDS was used for further analysis.

	**Mean Length**	**N50**	**Total**
**CDS**	416 bp	744 bp	149, 825
**Protein**	147 aa	277 aa	149, 825

Table S5. Statistics of expressed unigenes for each species, related to Figure 1. Reads from each species were independently mapped to Combined unigenes, and the number of mapped reads was normalized by Fragments Per Kilobase of exon model per Million mapped fragments (FPKM) method. The FPKM threshold was set as 0.3 in this study.

Sample	200-500 bp	500-1k bp	1k-2k bp	>2k bp	Mean Length	N50	Total
**HA**	51,145	18,820	14,031	11,127	876	1,622	95,123
**CD**	507,09	15,712	11,446	7,264	754	1,339	85,131
**HC**	52,328	21,776	19,402	15,069	972	1,757	108,575
**Hau**	26,232	12,122	10,662	7,068	949	1,626	56,084

Table S6. Statistics of expressed unigenes that contain coding sequence for all species used in this study, related to Figure 1.

Sample	200-500 bp	500-1k bp	1k-2k bp	>2k bp	Mean Length	N50	Total
**HA**	29,393	13,994	12,940	11,029	1,067	1,911	67,356
**CD**	28,015	12,477	11,024	7,235	934	1,615	58,751
**HC**	28,524	16,725	18,192	14,956	1,188	1,970	78,397
**Hau**	15,258	9,929	10,334	7,044	1,126	1,796	42,565

Table S7. Statistics of filtered unigenes that were correctly assembled, related to Figure 1 and 2. The unigenes of each species showed in Table S6 were screened against four local libararies (unigenes of HAC and Cis, full-length transcriptome of *C. deserticola* (CD_FL) and *H. ammodendron* (HA_FL)) using dual BLASTN (E value = $1e^{-10}$), and the union set of the blasted unigenes in four library were counted.

Sample	200-500 bp	500-1k bp	1k-2k bp	>2k bp	Mean Length	N50	Total(union)
**HA**	28,829	13,890	12,907	11,026	1,074	1,919	66,652
**CD**	26,407	12,432	11,018	7,234	952	1,631	57,091
**HC**	27,874	16,634	18,155	14,952	1,196	1,976	77,615
**Hau**	13,140	9,833	10,324	7,044	1,173	1,818	40,341

Table S8. Statistics for full-length transcriptome of CD_FL and HA_FL, related to Figure 1. Subread statistics.

Sample	Subreads(G)	Total number	Mean Length	N50
**CD_FL**	23.1	9,655,882	2,393	2,614
**HA_FL**	16.23	6,602,625	2,458	2,962

Table S9. Statistics of CD_FL and HA_FL, related to Figure 1. Circular Consensus Sequence (CCS) and Full-Length Non Chimera (FLNC) sequence.

Sample	CCS	5'-primer	3'-primer	Poly-A	Full length	FLNC	Mean Length
**CD_FL**	658,666	619,190	613,304	553,518	514,543	499,356	2,614
**HA_FL**	357,147	333,288	334,957	324,579	299,801	283,470	2,974

Table S10. Statistics of CD_FL and HA_FL, related to Figure 1. Consensus reads.

Sample	<1k bp	1k-2k bp	2k-3k bp	>3k bp	Mean Length	N50	Total
**CD_FL**	3,853	50,192	136,568	77,179	2,721	2,795	267,792
**HA_FL**	3,794	24,323	52,984	59,407	3,038	3,356	140,508

Table S11. Statistics of CD_FL and HA_FL, related to Figure 1. Non-Redundant Full-Length (FL_NR) reads.

Sample	<1k bp	1k-2k bp	2k-3k bp	>3k bp	Mean Length	N50	Total
**CD_FL**	2,340	31,582	66,617	64,382	2,937	3,111	164,921
**HA_FL**	2,022	11,464	28,740	47,022	3,319	3,832	89,248

Table S12. Confirmation of mobile direction for trans unigenes, related to Figure 1 and 2. The unigenes that were contained in both CD and HC were screened against Orobanchaceae or Chenopodiaceae sequences by dual BLASTN (E value = $1e^{-10}$). EST and mRNA sequences of two families were downloaded from NCBI database. Unigenes of GSE63970 and GSE93684 were downloaded from GEO database. Transcriptome of three Orobanchaceae species (OrAe, StHe, TrVe) were downloaded from Parasitic Plant Genome Project (PPGP) website (ppgp.huck.psu.edu/plants.php). The union set of the blasted unigenes in each library were counted.

Chenopodiaceae	EST	mRNA	HA_FL	GSE63970	GSE93684	Total
**14,810**	433	1,896	1,248	809	801	2,842
**2,569**	767	1,603	2,211	2,253	2,241	2,459
**Total**	1,200	3,499	3,459	3,062	3,042	5,301

Orobanchaceae	EST	mRNA	OrAe	StHe	TrVe	Total
**14,810**	6,537	267	8,245	6,550	7,503	9,521
**2,569**	545	30	686	579	635	906
**Total**	7,082	297	8,931	7,129	8,138	10,427

Table S13. List of proteomic data used in Orthogroup analysis, related to Figure 2.

Species	Accession No.	Source	Data Type	Notes
Amborella trichopoda	GCF_000471905.2	NCBI	Genome	sister group
Cinnamomum micranthum	GCA_003546025.1	NCBI	Genome	magnoliid dicot
Oryza sativa, japonica	Version 7.0	MSU	Genome	monocots
Zea mays	Zm-B73-REFERENCE-NAM -5.0	Ensemble	Genome	monocots
Sorghum bicolor	Sorghum_bicolor_NCBIv3	Ensemble	Genome	monocots
Aquilegia coerulea	GCA_002738505.1	NCBI	Genome	early diverging eudicots
Nelumbo nucifera	GCF_000365185.1	NCBI	Genome	early diverging eudicots
Prunus persica	GCF_000346465.2	NCBI	Genome	eurosids
Glycine max	GCF_000004515.6	NCBI	Genome	eurosids
Medicago truncatula	GCF_003473485.1	NCBI	Genome	eurosids
Arabidopsis thaliana	Non	TAIR10	Genome	eurosids
Carica papaya	GCF_000150535.2	NCBI	Genome	eurosids
Citrus sinensis	GCF_000317415.1	NCBI	Genome	eurosids
Gossypium raimondii	GCF_000327365.1	NCBI	Genome	eurosids
Theobroma cacao	GCF_000208745.1	NCBI	Genome	eurosids
Beta vulgaris	GCF_000511025.2	NCBI	Genome	Host, Chenopodiaceae
Spinacia oleracea	GCF_002007265.1	NCBI	Genome	Host, Chenopodiaceae
Chenopodium quinoa	GCF_001683475.1	NCBI	Genome	Host, Chenopodiaceae
Actinidia chinensis	GCA_003024255.1	NCBI	Genome	asterids
Helianthus annuus	GCF_002127325.2	NCBI	Genome	asterids
Daucus carota	ASM162521v1	Ensemble	Genome	asterids
Coffea canephora	GCA_900059795.1	NCBI	Genome	asterids
Nicotiana attenuata	GCF_001879085.1	NCBI	Genome	asterids
Solanum tuberosum	Stuberosum_v4.03	Phytozome	Genome	asterids
Cuscuta australis	GCA_003260385.1	NCBI	Genome	Holoparasitic, asterids
Ipomoea nil	GCF_001879475	NCBI	Genome	asterids
Olea europaea	GCF_002742605.1	NCBI	Genome	asterids
Mimulus guttatus	Mimulus guttatus v1.0	JGI	Genome	asterids
Sesamum indicum	GCF_000512975.1	NCBI	Genome	asterids
Handroanthus impetiginosus	GCA_002762385.1	NCBI	Genome	asterids
Phelipanche aegyptiaca	OrAeBC5	PPGP	Transcriptome	Holoparasitic, asterids
Phtheirospermum japonicum	GCA_014905375.1	NCBI	Genome	Hemiparasitic, asterids
Triphysaria versicolor	TrVeBC3	PPGP	Transcriptome	Hemiparasitic, asterids
Striga asiatica	GCA_008636005.1	NCBI	Genome	Hemiparasitic, asterids
Striga hermonthica	StHeBC3	PPGP	Transcriptome	Hemiparasitic, asterids
Haloxylon ammodendron	HA	This paper	Transcriptome	Host, Chenopodiaceae
Cistanche deserticola	CD	This paper	Transcriptome	Holoparasitic, asterids

Table S14. Statistics of lost genes in *C. deserticola* and six other parasitic plants, related to Figure 2. Orthogroups present in at least three of the five asterids species, *C. canephora*, *H. annuus*, *M. guttatus*, *S. indicum* and *S. tuberosum* were considered to be conserved across asterids, and the gene loss was assessed by comparing with the sequence of *M. guttatus*.

Species	Parasitsm	No. of Conserved Orthogroups	No. of Lost Orthogroups compared with *M. guttatus*	No. of Lost Genes compared with *M. guttatus*
Phtheirospermum japonicum	hemiparasitic	10, 761	520 (4.83%)	691
Triphysaria versicolor	hemiparasitic	10, 761	538 (5.00%)	810
Striga hermonthica	hemiparasitic	10, 761	587 (5.45%)	958
Striga asiatica	hemiparasitic	10, 761	784 (7.29%)	1, 288
Phelipanche aegyptiaca	holoparasitic	10, 761	1375 (12.78%)	2, 126
Cuscuta australis	holoparasitic	10, 761	1486 (13.81%)	2, 951
Cistanche deserticola	holoparasitic	10, 761	1720 (15.98%)	3, 095

Table S15. Length distribution of trans and no-trans unigenes, related to Figure 1 and 2. HC_Trans, unigenes that mobiled from *H. ammodendron* to *C. deserticola*; CD_Trans, unigenes that mobiled from *C. deserticola* to *H. ammodendron*; Homolog, homologs between Chenopodiaceae and Orobanchaceae; HC_no_Trans and CD_no_Trans, unigenes that didn't transfer between *H. ammodendron* and *C. deserticola*; CD_unique, unigenes of *C. deserticola* except *H. ammodendron* mobile unigenes; HC_unique, unigenes of *H. ammodendron* except *C. deserticola* mobile unigenes.

Sample	200-500 bp	500-1k bp	1k-2k bp	>2k bp	Mean Length	N50	Total
**HC_Trans**	265	426	830	849	1,777	2,326	2,370
**CD_Trans**	423	1,352	3,320	2,401	1,728	2,066	7,496
**Homolog**	108	282	946	1,595	2,390	2,846	2,931
**HC_no_Trans**	27,186	14,856	14,005	11,702	1,117	1,927	67,749
**CD_no_Trans**	25,719	10,654	6,868	3,984	788	1,284	47,225
**CD_unique**	26,142	12,006	10,188	6,385	917	1,569	54,721
**HC_unique**	27,451	15,282	14,835	12,551	1,140	1,954	70,119
